# Association between climatic variables and cardiovascular hospitalizations in Brazil: An ecological study

**DOI:** 10.1371/journal.pgph.0005294

**Published:** 2026-07-29

**Authors:** Bruna Dóris, Gabriel Savogin Andraus, Rajesh da Silva Seunaraine, Débora Nabor de Cássia Silva, Natália da Silva Teixeira, Bruno Siegel Guerra, Thyago Proença de Moraes, Gustavo Lenci Marques

**Affiliations:** 1 Universidade Federal do Paraná, Postgraduate Program in Internal Medicine and Health Sciences, Curitiba, Paraná, Brazil; 2 Pontifícia Universidade Católica do Paraná, Curitiba, Paraná, Brazil; 3 MUNAI, Curitiba, Paraná, Brazil; PLOS: Public Library of Science, UNITED STATES OF AMERICA

## Abstract

Cardiovascular disease is the leading cause of death worldwide, and external factors such as ambient temperature can predispose to cardiovascular events. Brazil, a country with continental dimensions and diverse climates, lacks a recent study comparing cardiovascular hospitalization rates with climatic data across the Brazilian states. This study aimed to describe and relate the hospitalization counts of cardiovascular events, including acute myocardial infarction, stroke, and heart failure, to climatic data. Data were obtained from the Department of Informatics of the Unified Health System and the National Meteorology Institute from 2010 to 2024. We recorded a total of 2,917,900 hospitalizations for cardiovascular diseases across 103 cities in Brazil where complete climatic data were available. The hospitalization rates per 100,000 inhabitants ranged from 0.007 in the state of Maranhão to 0.023 in Rio Grande do Sul. We observed a lower relative risk of hospitalization for cardiovascular diseases in regions with a higher ambient temperature, with the temperature range up to 19°C having the highest risk. No difference in hospitalization risk was observed between the temperate and tropical climate regions. This study highlights the importance of public measures for cardiovascular event prevention during periods of lower temperature throughout the year.

## Introduction

Cardiovascular disease is the leading cause of death in Brazil and worldwide, and its prevalence has increased in recent decades due to population aging [1–3]. External factors, such as pollution levels, stress, and climate, can influence the cardiovascular system. The latter has been widely studied and classically described in literature, with cardiovascular mortality rates expected to vary according to seasonal and daily temperature fluctuations. However, published studies on this topic have yielded divergent results, with some indicating a higher risk associated with heatwaves [4–6] and others suggesting a higher risk with exposure to lower temperatures [7–12].

The evidence supporting the risk of cardiovascular events with lower temperatures is more robust. It is well established that exposure to lower temperatures induces sympathetic hyperactivity, thereby increasing the cardiac workload. Furthermore, increased platelet activation and hemoconcentration have been observed, leading to a heightened risk of atherothrombosis [8,12–14]. Research has shown that these effects can persist for up to four weeks following exposure to cold temperatures [8,10].

In this context, recent multicenter studies evidenced that approximately 8–9% of annual cardiovascular deaths worldwide are attributable to exposure to non-optimal ambient temperatures. Cold exposure accounts for the majority of temperature-related mortality (8.2%), whereas heat exposure contributes a comparatively smaller proportion (0.66%) [10,15]. A German ecological study compared daily acute myocardial infarction (AMI) rates from 1987 to 2014 with average ambient temperatures. The results revealed that temperatures below 18·4 °C were associated with a higher relative risk of AMI [16]. An American study analyzing data from 1979 to 2002 in Minnesota found seasonal variations in sudden cardiac death, with a peak in winter and a nadir in summer [11]. Another retrospective study demonstrated that winter was associated not only with higher infarction rates, but also with more extensive and severe events [17]. Japanese researchers found a correlation between lower temperatures and an increased risk of hospitalization for cardiovascular reasons, particularly in the older adults [18].Some studies suggest that the relationship between cardiovascular mortality and ambient temperature follows a U-shaped curve, with temperatures between 15 °C and 25 °C associated with the lowest mortality rates [9,10,19,20].

However, establishing this association can be particularly challenging in countries like Brazil, given its continental territorial extent of approximately 8.5 million km^2^, which contains substantial climatic heterogeneity [21]. Consequently, there are limited number of studies about this topic in the country. A 2019 Brazilian study evaluated the daily temperatures and myocardial infarction mortality in six capitals in different regions, climates, and socioeconomic conditions between 1996 and 2013. The results were consistent with those of international studies, which showed higher AMI rates in winter in locations with greater temperature amplitudes. However, in cities closer to the equator, myocardial infarction mortality did not exhibit a significant relationship with temperature [9]. Another national study involving capitals from distinct regions found that the risk of hospital admission for cardiovascular reasons was lower during summer [6]. While these studies had significant impacts, they had limitations, such as focusing solely on myocardial infarction mortality in the capital or evaluating only periods of higher temperatures.

Brazil comprises two predominant climate patterns: a temperate climate, predominantly observed in the Southern region, and a tropical climate, which characterizes most of the national territory. The Amazon region represents a distinct climatic setting, characterized by persistently high humidity levels and classified as equatorial climate, a subtype of tropical climate [22]. To date, no studies have systematically compared hospitalization rates for cardiovascular events across the different climatic zones within Brazil.This study aimed to describe the hospitalization rates of cardiovascular events (including AMI, stroke and arrythmias) in Brazil from 2010 to 2024 and its association with daily average temperatures. Additionally, it investigated differences in the hospitalization counts of cardiovascular events between the temperate and tropical climate regions in the country.

## Materials and methods

### Study design

This is an ecological study based on the analysis of extensive national records with open data for public consultation. According to the Conselho Nacional de Saúde Resolution 510/2016, because this research used publicly accessible information databases in accordance with Law No. 12,527 of November 18, 2011, whose aggregated information does not allow individual identification, approval by the Research Ethics Committee/National Research Ethics Committee was not required.

### Study setting

The study was conducted in Brazil, a country of continental dimensions encompassing basically two climate zones (tropical and temperate). The analysis evaluated data from multiple Brazilian municipalities over a 14.5-year period, from January 2010 to June 2024.

### Study population, sample size, and sampling method

The study population consisted of all residents in the included Brazilian cities who were hospitalized for cardiovascular events during the study period. Due to the ecological nature of the study, a non-probability purposive sampling method was applied, encompassing all available national health and meteorological records that met the inclusion criteria. The final sample size comprised a total of 2,917,900 cardiovascular hospitalizations across 103 Brazilian cities.

### Inclusion and exclusion criteria

Brazil has an open-access health data system, known as Department of Informatics of the Unified Health System (DATASUS), which provides nationwide health data in a publicly accessible manner. The number of cardiovascular hospitalizations was directly extracted from the national system to obtain epidemiological data. For this analysis, all hospitalization rates by residential municipality from January 2010 to June 2024 were included for the following International Classification of Diseases (ICD)-10 codes: acute myocardial infarction (ICD-10: I21, I22), other ischemic heart diseases (ICD-10: I20, I23-I25), heart conduction disorders and cardiac arrhythmias (ICD-10: I44-I49), heart failure (ICD-10: I50), cerebral infarction (ICD-10: I63), and stroke (ICD-10: I64).

Meteorological data were automatically extracted from the National Meteorological Institute (INMET). Data from all Brazilian meteorological stations, including hourly average temperatures, were obtained from January 2010 to June 2024. All extracted data were converted from their native format to the comma-separated value (CSV) format, generating an. csv file for better interpretation and analysis using R.Cities were excluded from the analysis if the total number of cardiovascular events was lower than the total number of observation days (5,296 days), as this would compromise the convergence and stability of the statistical models. Furthermore, cities with intermittent or sparse meteorological or hospitalization data were removed to avoid compromising the validity of the estimated lag effects and seasonal trends.

### Statistical analysis

All statistical analyses, as well as the creation of graphs and tables, were performed using R software (version 4.0.0 or higher), with intensive use of the *dlnm*, *mvmeta*, *splines*, *mgcv*, and *tidyverse* packages.

Initially, publicly available data from the meteorological system and DATASUS were processed, cleaned, and categorized. Hourly meteorological data were obtained from the National Meteorological Institute (INMET) surface weather stations. Stations were geographically allocated to cities using spatial information from the *geobr* package, which allowed for the accurate identification of municipal boundaries and the disambiguation of capital cities with identical names. In municipalities with multiple active stations, data were pooled. The daily mean temperature (DAILY_MEAN_TEMP) was calculated as the arithmetic mean of all valid hourly minimum and maximum temperature readings recorded across all stations within the city for that specific day. Quality control excluded intermittent or sparse data to ensure the integrity of the modeling.

Given the non-normal nature of the daily cardiovascular hospitalization counts (which exhibited overdispersion), these data are reported as total sums and medians (minimum – maximum), while daily mean temperatures are summarized using means (± standard deviation) due to their approximately normal distribution. Qualitative variables are expressed as absolute numbers (n) and percentages (%).

We modeled the association between daily mean temperature and the incidence of cardiovascular hospitalizations across different Brazilian cities using a standard two-stage analytical approach, as recommended for multi-city environmental epidemiology studies [10,23]. In the first stage, the association was assessed for each city individually; in the second stage, the city-specific results were combined through a multivariate meta-analysis [23].

### First stage

A generalized linear model (GLM) with a quasi-Poisson family was fitted to the daily hospitalization counts to account for overdispersion. To model the non-linear and delayed effects of temperature, a distributed lag non-linear model (DLNM) was employed. The primary model equation was defined as:


$$\begin{array}{c} og\left(E\left[Y_{t}\right]\right)=\;\alpha +\;cb\left(temp_{t},\;lag\right)+\;ns(time,\;df=\text{7}\text{2})+\;\gamma dow_{t} \end{array}$$

Where $E\left[Y_{t}\right]\;$ represents the expected cardiovascular hospitalization count on day t, and α is the model intercept. The term $cb\left(temp_{t},\;lag\right)\;$ is the cross-basis matrix generated by the DLNM framework. For the predictor space (temperature), we used a quadratic B-spline (degree = 2) with three internal knots placed at the 25th, 50th, and 75th percentiles of each specific city’s temperature distribution. For the lag-response relationship, we applied a natural cubic B-spline with a maximum lag of 7 days and three internal knots placed at equally spaced values on a logarithmic scale. The term ns(time, df=72)  represents a natural cubic spline with 72 degrees of freedom (equivalent to 5 df per year over the 14.5-year period) to control long-term trends and seasonality. Finally, dow_t is a categorical variable adjusting for the day-of-the-week effect.

From the estimated effect curve of each city, the Minimum Morbidity Temperature (MMT) was identified. The Relative Risks (RR) were subsequently calculated using the specific MMT as the centering reference point (RR = 1.0). The estimated MMT and corresponding RR values for each city are provided in S2 Data.

### Second stage

The city-specific coefficients and covariance matrices extracted in the first stage were pooled using a multivariate meta-analysis with Restricted Maximum Likelihood (REML) estimation. The baseline meta-analytical model can be summarized as:


$$\begin{array}{c} {\left\{\beta \right\}}_{i}\sim N\left(\mu ,\;S_{i}+\;\Sigma \right) \end{array}$$

Where${\;\left\{\beta \right\}}_{i}$ is the vector of coefficients for city i, μ is the overall pooled exposure-response vector, $S_{i}$ is the within-city estimation variance, and Σ is the between-city (heterogeneity) variance matrix.

This pooling process was repeated for macro-regions (North, Northeast, Central-West, Southeast, and South) and for Brazil as a whole. To evaluate the effect of climate type, we conducted a meta-regression incorporating the climate category as a moderator, effectively modeling μ_i = μ_0 + X_i * θ, where X_i indicates if city i is “Temperate” or “Tropical”.

Notably, the city of Belém was explicitly excluded from the pooled analysis of the North Region. Belém’s equatorial climate exhibited extremely low thermal variability (standard deviation = 1.00°C). Applying the standardized quadratic spline specification to this low-variance data caused numerical instability and biologically implausible estimates. Additionally, we identified a nominal inconsistency, as there are two cities in the country named Belém. To maintain methodological consistency across the multi-city pooling, Belém was excluded from the main meta-analysis, although sensitivity analyses using simplified linear models confirmed that this exclusion did not bias the regional estimates.

## Results

**Fig 1:**
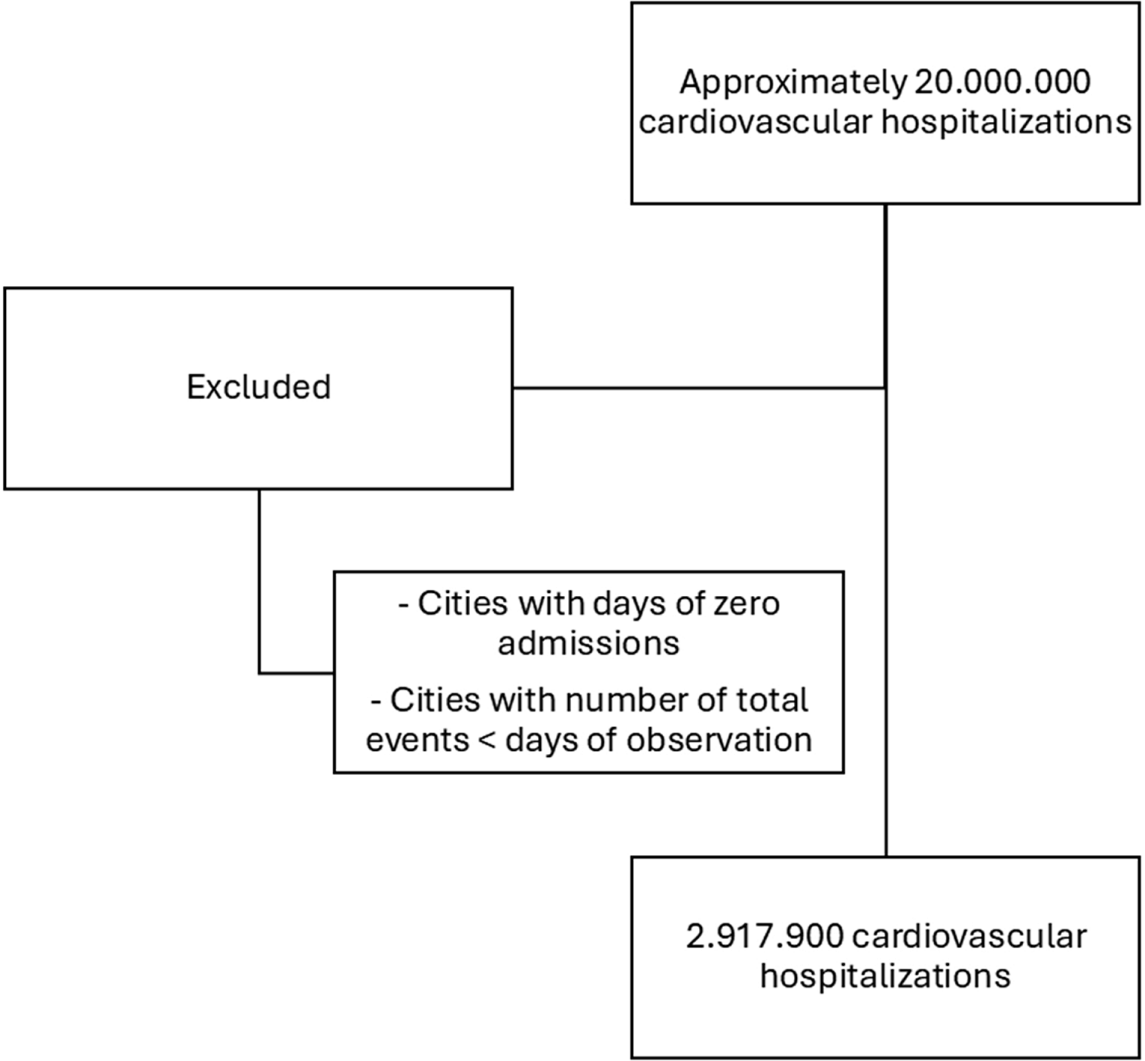
Flowchart of the Department of Informatics of the Unified Health System (DATASUS) data collection.

Between January 2010 and June 2024, 2,917,900 hospitalizations for cardiovascular diseases were registered in Brazil in 103 cities with fully available climate data. The total number of hospitalizations in Brazil was significantly higher (approximately 20,000,000 cardiovascular-related hospitalizations). However, these data were excluded because not all Brazilian cities had complete climatic data (Fig 1). Additionally, cities with fewer cardiovascular hospitalizations during the analysis period than on the days of observation (5296 days) were also excluded. The complete data on cardiovascular hospitalizations and mean temperature for all 103 cities included in the analysis during the study period are avaiable in S1 Data. Table 1 summarizes the number of events, population size, mean temperature, and climate classification for each of these cities. All state capitals were included in this study.

**Table 1:** List of cities categorized by their respective states and regions of Brazil with the number of cardiovascular events registered on the period of January 2010 to June 2024, population (according to last census 2022 from Instituto Brasileiro de Geografia e Estatística – IBGE – available on https://www.ibge.gov.br/cidades-e-estados), mean temperature and assigned climate type.

Southern region	Number of events	Population	Mean temperature (ºC)	Climate type
**STATE OF PARANA**		**11.824.665**		
Curitiba	79.306	1.773.718	18.3	Temperate
Foz do Iguaçu	10.269	285.415	22.07	Temperate
Maringá	23.133	409.657	22.9	Temperate
TOTAL	**112.708**			
**STATE OF SANTA CATARINA**		**8.058.411**		
Florianópolis	18.013	537.211	21.35	Temperate
Itajaí	23.729	264.054	20.61	Temperate
Lages	7.800	172.458	16.66	Temperate
Xanxerê	18.682	136,73	18.8	Temperate
TOTAL	**68.224**			
**STATE OF RIO GRANDE DO SUL**		**11.229.915**		
Bage	7.584	117.938	18	Temperate
Passo Fundo	56.499	206.215	18.06	Temperate
Pelotas	7.210	325.685	18.58	Temperate
Porto Alegre	148.337	1.332.845	20.1	Temperate
Rio Grande	22.049	191.900	18.78	Temperate
Santa Maria	9.688	271.735	19.47	Temperate
Uruguaiana	5.324	120.819	19.92	Temperate
Tramandaí	7.175	56.430	19.98	Temperate
TOTAL	263.866			
**Southeast region**	**Number of events**	**Population**	**Mean temperature (ºC)**	**Climate type**
**STATE OF MINAS GERAIS**		**21.322.691**		
Barbacena	20.033	125.317	18.66	Tropical
Belo Horizonte	118.067	2.315.560	19.95	Tropical
Curvelo	5.788	80.665	23.19	Tropical
Diamantina	9.252	47.702	18.62	Tropical
Divinópolis	7.102	231.091	22.09	Tropical
Formiga	5.305	68.248	21.71	Tropical
Governador Valadares	19.954	257.171	24.44	Tropical
Juiz de Fora	44.169	540.756	19.37	Tropical
Montes Claros	46.167	414.240	24.03	Tropical
Muriaé	23.236	104.108	23.28	Tropical
Passos	13.017	111.939	21.58	Tropical
Patos de Minas	2.903	159.235	22.06	Tropical
Sete Lagoas	9.060	227.397	22	Tropical
São Joao Del Rei	6.048	94.468	19.82	Tropical
São Sebastiao do Paraiso	5.776	71.796	21.21	Tropical
Teófilo Otoni	7.194	137.418	23.3	Tropical
Uberaba	10.922	337.836	22.79	Tropical
Uberlândia	31.690	761.835	23.53	Tropical
Varginha	17.743	143.676	20.4	Tropical
TOTAL	403.426			
**STATE OF SAO PAULO**		**44.411.238**		
Barretos	9.077	122.485	23.11	Tropical
Bauru	22.006	379.146	22.01	Tropical
Franca	15.455	352.536	21.73	Tropical
Itapeva	6.524	89.728	19.98	Tropical
Jales	6.069	48.776	24.74	Tropical
Marilia	9.117	237.627	22.79	Tropical
Ourinhos	6.680	103.970	22.24	Tropical
Piracicaba	16.493	423.323	22.01	Tropical
Presidente Prudente	27.239	225.668	23.97	Tropical
Sorocaba	34.631	723.682	20.91	Tropical
São Carlos	16.000	254.857	21.19	Tropical
São Paulo	450.734	11.451.999	20.56	Tropical
Taubaté	15.956	310.739	21.1	Tropical
Tupã	3.159	63.928	24.02	Tropical
Votuporanga	9.619	96.634	24.5	Tropical
TOTAL	648.759			
**STATE OF RIO DE JANEIRO**		**16.055.174**		
Duque de Caxias	13.921	808.161	23.05	Tropical
Macaé	7.505	246.391	23.55	Tropical
Niterói	5.578	481.749	24.83	Tropical
Nova Friburgo	14.295	189.939	17.08	Tropical
Rio de Janeiro	69.119	6.211.223	23.23	Tropical
São Gonçalo	22.210	896.744	27.54	Tropical
TOTAL	132.628			
**STATE OF ESPÍRITO SANTO**		**3.833.712**		
Linhares	14.371	166.786	24.36	Tropical
São Mateus	6.779	123.752	24	Tropical
Vila Velha	13.986	467.722	23.92	Tropical
Vitoria	22.898	322.869	24.51	Tropical
TOTAL	58.034			
**Northeast region**	**Number of events**	**Population**	**Mean temperature**	**Climate type**
**STATE OF SERGIPE**		**2.291.077**		
Aracaju	24.453	602.757	26.71	Tropical
**STATE OF ALAGOAS**		**3.220.104**		
Arapiraca	9.944	234.696	24.88	Tropical
Maceió	47.693	957.916	25.54	Tropical
TOTAL	57.637			
**STATE OF CEARA**		**9.233.656**		
Barbalha	7.307	75.033	26.36	Tropical
Fortaleza	132.794	2.428.708	27.45	Tropical
Sobral	25.009	203.023	27.82	Tropical
TOTAL	165.110			
**STATE OF MARANHÃO**		**6.775.152**		
Imperatriz	10.778	273.110	27.36	Tropical
São Luis	37.004	1.037.775	26.91	Tropical
TOTAL	47.782			
**STATE OF BAHIA**		**14.850.513**		
Barreiras	6.401	159.734	25.81	Tropical
Feira de Santana	13.732	616.272	24.78	Tropical
Ilheus	6.865	178.649	23.71	Tropical
Salvador	101.484	2.417.678	25.81	Tropical
Vitoria da Conquista	18.247	370.879	20.61	Tropical
TOTAL	146.729			
**STATE OF RORAIMA**		**716.793**		
Boa Vista	8.916	413.486	27.57	Tropical
**STATE OF PARAIBA**		**4.145.000**		
Campina Grande	17.872	419.379	23.81	Tropical
João Pessoa	35.625	833.932	26.67	Tropical
Patos	4.771	103.165	28.44	Tropical
TOTAL	58.268			
**STATE OF PERNAMBUCO**		**9.058.931**		
Caruaru	18.149	378.048	22.51	Tropical
Petrolina	9.311	386.791	27.31	Tropical
Recife	154.498	1.488.920	25.86	Tropical
TOTAL	181.958			
**STATE OF RIO GRANDE DO NORTE**		**3.446.071**		
Mossoró	16.536	264.577	27.51	Tropical
Natal	41.966	751.300	26.79	Tropical
TOTAL	58.502			
**STATE OF PIAUI**		**3.375.646**		
Parnaíba	6.067	162.159	27.33	Tropical
Picos	5.056	83.090	28.72	Tropical
Teresina	46.830	866.300	28.07	Tropical
TOTAL	57.953			
**Northern region**	**Number of events**	**Population**	**Mean temperature**	**Climate type**
**STATE OF TOCANTINS**		**1.511.460**		
Araguaína	9.714	171.301	25.92	Tropical
Palmas	14.606	302.692	27.55	Tropical
TOTAL	24.320			
**STATE OF RORAIMA**		**716.793**		
Boa Vista	8.916	413.486	27.57	Tropical
**STATE OF RONDONIA**		**1.581.196**		
Cacoal	4.421	86.887	26.68	Tropical
Porto Velho	11.097	460.434	26.67	Tropical
TOTAL	15.518			
**STATE OF AMAPA**		**802.837**		
Macapá	7.908	442.933	27.37	Tropical
**STATE OF AMAZONAS**		**4.281.209**		
Manaus	47.130	2.063.689	27.78	Tropical
**STATE OF ACRE**		**880.631**		
Rio Branco	7.884	364.756	25.72	Tropical
**STATE OF PARA**		**8.120.131**		
Santarém	6.354	331.942	26.7	Tropical
Belém	50.853	1.303.403	26.92	Tropical
TOTAL	57.207			
**Central-west region**	**Number of events**	**Population**	**Mean temperature**	**Climate type**
**BRASILIA**	58.276	**2.817.381**	21.56	Tropical
**STATE OF MATO GROSSO DO SUL**		**2.901.895**		
Campo Grande	46.272	898.100	24.12	Tropical
Dourados	10.961	243.367	23.38	Tropical
TOTAL	57.233			
**STATE OF GOIAS**		**7.056.495**		
Catalão	6.853	114.427	23.31	Tropical
Goiânia	118.238	1.437.366	23.85	Tropical
TOTAL	125.091			
**STATE OF MATO GROSSO**		**3.836.399**		
Cuiabá	22.591	650.877	27.19	Tropical
Rondonopolis	11.366	244.911	25.7	Tropical
TOTAL	33.957			

We calculated the hospitalization rates of cardiovascular diseases in each state during this period based on the number of hospitalizations and number of inhabitants in the state according to the current Instituto Brasileiro de Geografia e Estatística registration for 2024. The lowest incidence was in the northeastern region (0.007 in the state of Maranhão), whereas the highest was in the southern region (0.023 in the state of Rio Grande do Sul). The complete hospitalization counts by state is provided in S1–S5 Tables.

**Fig 2:**
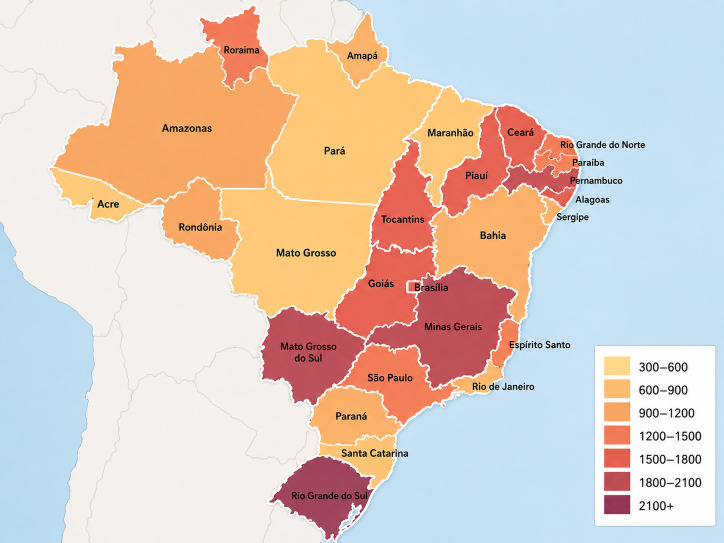
Rates of cardiovascular hospitalizations per 100,000 residents from 2010-2024 for each state in Brazil.

We mapped the hospitalization rates due to cardiovascular diseases per 100,000 inhabitants in each Brazilian state, as illustrated in Fig 2.

**Fig 3:**
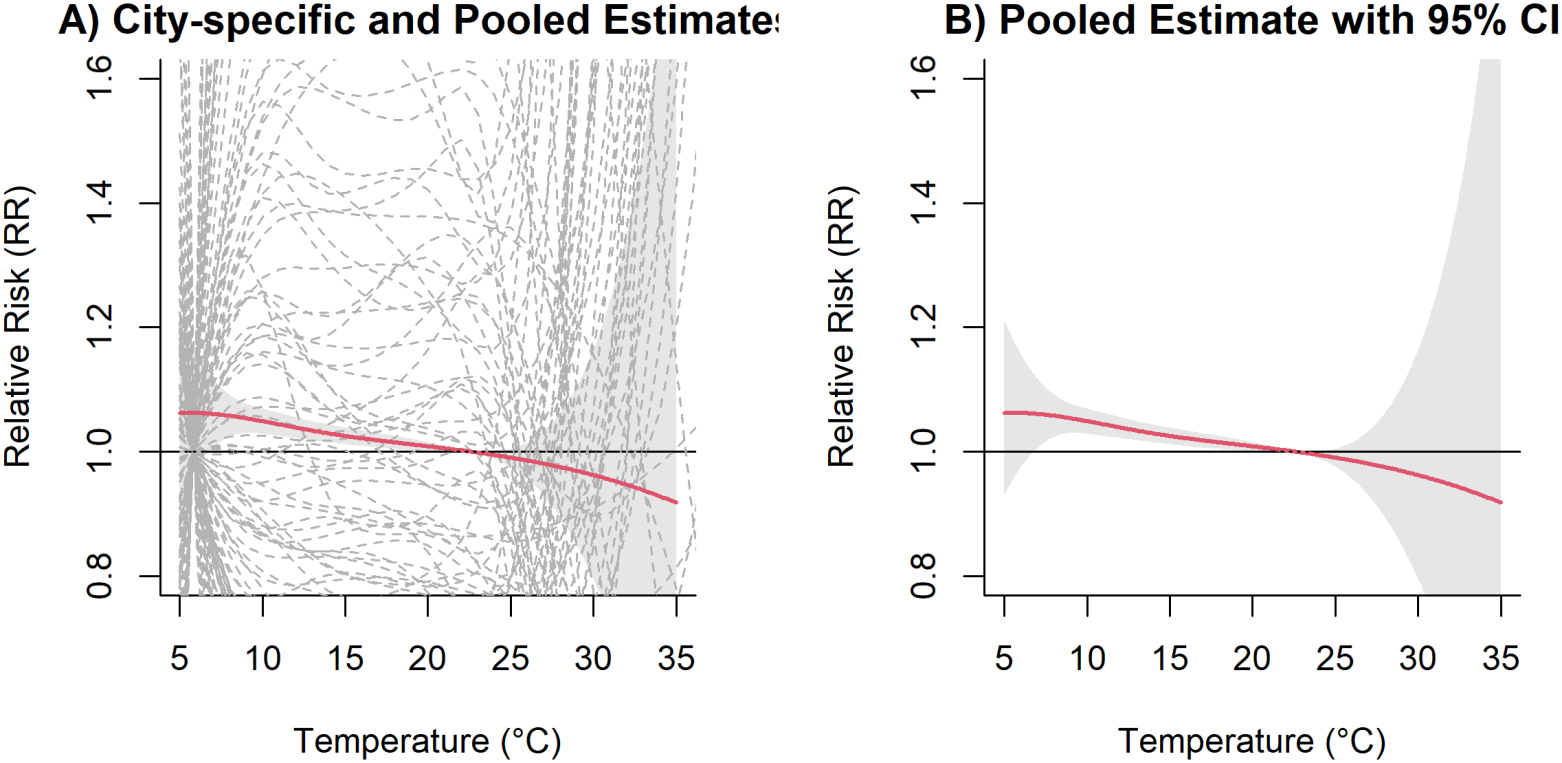
Relative risk of cardiovascular hospitalization according to daily median temperature in Brazil from 2010 to 2024. RR, relative risk; CI, confidence interval.

We plotted the data comparing the daily median temperature and the relative risk of cardiovascular hospitalization (Fig 3).

In the lowest temperature range (between 5 °C and 8 °C), there was a trend towards a higher rates of hospitalizations. Because few Brazilian cities reach lower temperatures, the confidence interval of the relative risk was broad and included 1.0. In this zone, the relative risk of hospitalization was 1.05, with the 95% confidence interval decreasing as the temperature increased: [0.86–1.28] at 5°C and [0.99–1.12] at 8°C.

Between temperatures of 9°C to 18°C, we observed a higher relative risk of hospital admission, with the risk increasing as the temperature decreased: at 9°C, the relative risk was 1.05 [1.01–1.09], at 14°C it was 1.03 [1.02–1.04], and at 18°C it was 1.01 [1.01–1.02].

From 19°C to 24°C, we observed a neutral relationship regarding hospitalization rates and ambient temperature. In our study, this range represents the optimal temperature range in Brazil.

Above 25°C, we observed a trend of a protective effect from heat concerning cardiovascular hospitalizations. As the temperature increased, the relative risk of hospitalization decreased: the risk was 0.99 [0.99–1.0] at 25°C, 0.97 [0.93–1.02] at 30°C, and 0.94 [0.69–1.28] at 35°C. In parallel with the lower temperature extremes, since few cities had sufficient data for daily medium temperatures above 25°C, the confidence interval crossed 1.0.

**Fig 4:**
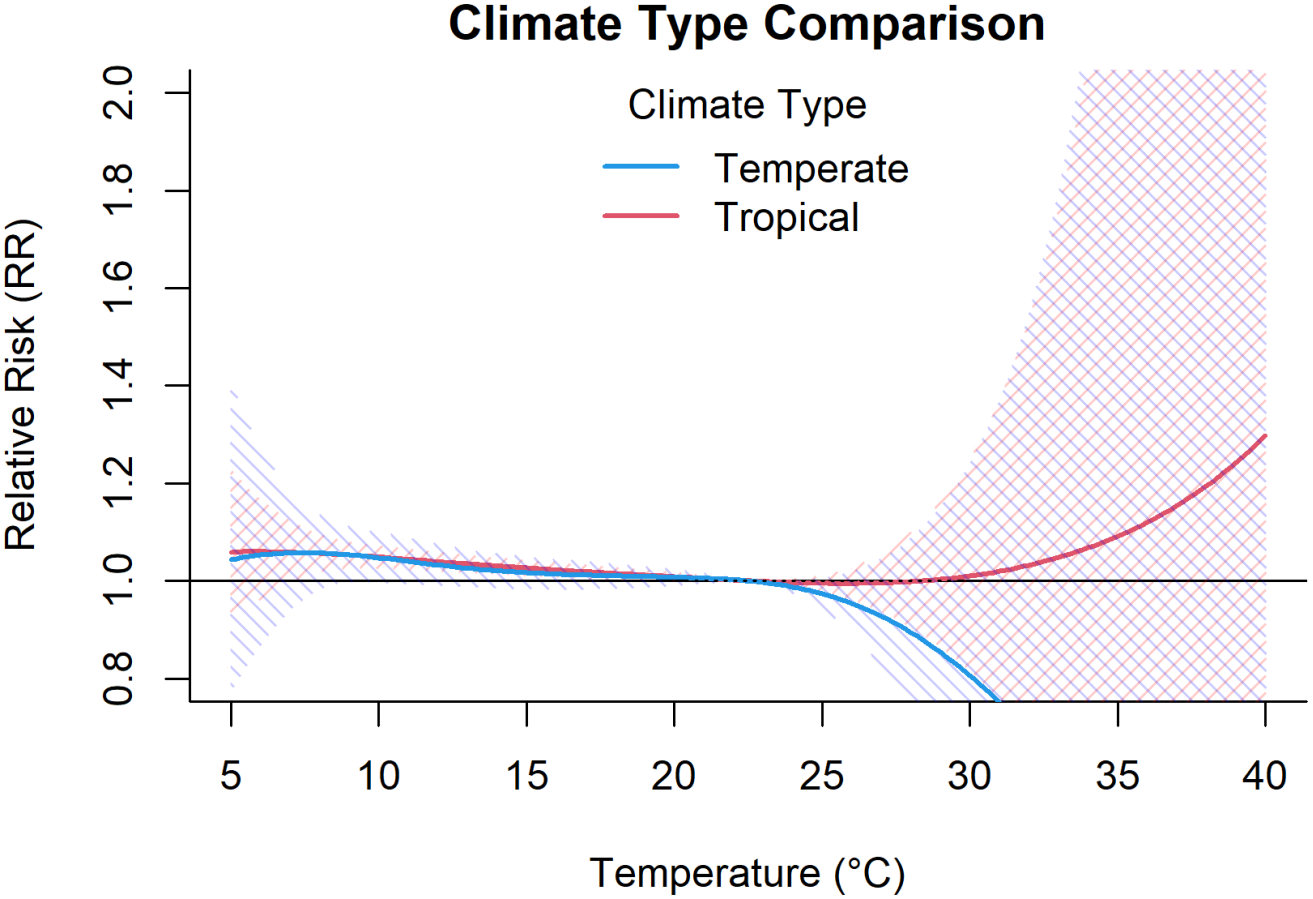
Relative risk of cardiovascular hospitalization according to the type of climate in Brazil during the period of 2010–2024. Shading indicates 95% confidence interval (CI). RR, relative risk.

As Brazil is a country of continental dimensions and most studies on the subject involve temperate climate countries, we compared the regions of temperate climate with those with tropical climate (Fig 4).

No significant difference in hospitalization rates for cardiovascular diseases was observed between the temperate and tropical climate regions. We noted a tendency for a higher hospitalization risk in both climates at lower temperatures. Between 20°C and 24°C, ambient temperature had a neutral effect on hospitalization incidence. Above 25°C, a trend toward a lower risk of hospitalization was observed. The confidence intervals increased with increasing temperature, probably because of the smaller amount of data at extreme temperatures.

## Discussion

To the best of our knowledge, this is the largest study conducted in Brazil that associates hospitalization for cardiovascular diseases with mean daily ambient temperature. Previous national studies have related climate data to cardiovascular mortality or have limitations, such as focusing on a specific cardiovascular disease, restricted time periods, or only covering capital cities.

The relationship between cardiovascular events and ambient temperature has been firmly established at lower temperatures, and our findings align with those reported in the literature. Decreased body temperature leads to vasoconstriction, increased blood pressure, heightened skeletal muscle tone for heat conservation, and an increased concentration of vasoconstrictor peptides. Consequently, the cardiac workload increases. Moreover, there may be greater crystallization of atherosclerotic plaques, which increases the risk of plaque rupture [1–6].

Our study encompassed various cardiovascular diseases and highlighted the association between hospitalization due to cardiovascular diseases and temperatures below 19°C. This finding is similar to that reported in a germany study, where the risk of hospitalization due to heart attack was higher until a temperature of 18·4°C [16]. In Japan, a study evaluated the tomographic characteristics of coronary plaques responsible for heart attacks in 202 patients and found a higher incidence of plaque rupture at lower temperatures. The average temperature at the peak of heart attacks was 16.5°C, with a higher risk of myocardial infarction between 10°C and 20°C [24].

Although the association between ambient temperature and other cardiovascular diseases in the literature is noteworthy, it is less pronounced than that with coronary artery disease [25,26]. An ecological study conducted in Taiwan analyzed data from 2012 to 2019 and found higher rates of first decompensation for heart failure during the winter months, especially in patients aged > 60 years with at least one comorbidity [27]. A multinational study exploring the association between cardiovascular event mortality in individuals over 40 years of age and ambient temperature in 27 countries. They found that, among all cardiovascular causes of death, the highest burden from extreme hot temperatures was seen for heart failure [23]. In contrast, it is well stablished that hospitalization rates for heart failure decompensation are higher in winter; however, this also occurs alongside higher rates of respiratory infections [28,29]. A multinational ecological study estimated that for every 1,000 deaths from stroke, nine were attributed to extreme cold and 1.6 to extreme heat [23]. Studies on stroke etiology are inconsistent, but generally, there is a greater risk of events of both etiologies (ischemic and hemorrhagic) at temperature extremes [25,30–32]. As cardiac arrhythmia is a broad term that encompasses various pathologies, studies on this topic are divergent [23,26]. There is a possible association between mortality from arrhythmias and lower temperatures but apparently not with higher temperatures [23]. This may be attributed to misclassification since a ventricular arrhythmia may result from cardiac ischemia [23,33]. Some studies have indicated an association between atrial fibrillation and lower temperatures; however, the evidence is weak [34,35].

We believe that in our study, hospitalizations for cardiovascular diseases as a whole were driven by coronary events. As our objective was to assess cardiovascular diseases, individual analyses of each ICD code were not conducted.

The number of coronary events over a 28-year period in Germany has been assessed, with stratification into two periods: 1987–2000 and 2001–2014. Similar to our study, cold was associated with higher rates of heart attack in both periods. Interestingly, in the more recent period, there was a greater influence of heat on these hospitalizations, and a warmer climate could be considered a potentially preventable trigger of heart attacks [16].

Increased body temperature leads to dehydration and volume depletion, activating the sympathetic system, causing tachycardia and subsequently increasing the cardiac workload. Hemoconcentration also contributes to hypercoagulability, which increases the risk of thrombosis and heart attacks [19,26].

An ecological study found that of the total cardiovascular deaths worldwide from 2000 to 2019, 8.86% were caused by a non-optimal ambient temperature (high or low). Of these, only 0.66% were related to excessive heat. With climate change, the risk of adverse cardiovascular outcomes may increase with warmer climates [15,26]. However, we did not observe this effect in our study. The number of hospitalizations for cardiovascular diseases in Brazil reduced as ambient temperature increased, with heat potentially having a protective effect when temperatures exceeded 20 °C.

There were no statistically significant differences between the data for tropical and temperate cities. However, temperate climate regions showed a tendency toward a “U-shaped” curve, similar to that observed in the German study after the year 2000 and other reports on cardiovascular mortality [9,10,14,19,26,36].

**Fig 5:**
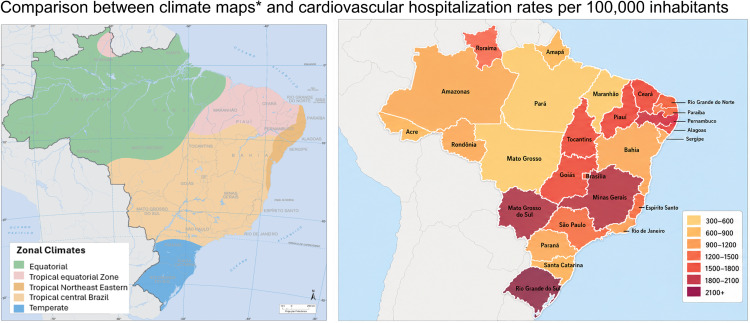
Comparison between climate maps* and rates of hospitalization due to cardiovascular diseases per 100,000 inhabitants in Brazil. *Adapted from Instituto Brasileiro de Geografia e Estatística [37].

Our study is the first one to compare regions with tropical climates with those with temperate climates in the same country. As shown in Fig 5, equatorial climate regions (a subtype of tropical climates) have the lowest rates of hospitalization for cardiovascular diseases. We can formulate a hypothesis regarding the humidity of the Amazon rainforest. However, humidity data were not assessed in this study. In the South, which is the only temperate region of the country, the state with the highest counts of cardiovascular hospitalization was Rio Grande do Sul. The eating habits in this region involve a diet high in fat; however, because our study did not evaluate this type of exposure, we cannot attribute it as a cause of more cardiovascular events.

The effects of temperature on the cardiovascular system vary according to the socioeconomic conditions. Populations with poorer socioeconomic conditions are more exposed to the effects of ambient temperature than are populations with better conditions [20,26,36]. This underscores the importance of public health measures to reduce the risk of cardiovascular hospitalization.

As previously mentioned in this article, the cardiovascular system is influenced by several factors beyond ambient temperature, including humidity, lifestyle habits, air pollution, and socioeconomic conditions [38,39]. The objective of our study was to evaluate ambient temperature exclusively, as data regarding the other factors are not widely available; however, this limitation provides an opportunity for future investigations.

The effect of temperature on the cardiovascular system does not necessarily imply immediate repercussions. There can be a time lag in exposure to a certain temperature that culminates in a cardiovascular event. It is estimated that exposure to heat can have consequences on the cardiovascular system within 3 days, whereas exposure to cold can exert an effect on the cardiovascular system in a more delayed manner, from 7 to 21 days [10,19,20]. In our study, we calculated a 7-day exposure period (high and low); however, the 7-day lag did not significantly influence hospitalization rates.

Under the current climate change scenario, this topic has gained importance as a public health measure for reducing cardiovascular morbidity and mortality. Public financial incentives for better home infrastructure, awareness campaigns, and increased financial support for health institutions focusing on cardiovascular treatment during extreme temperatures are examples of governmental measures that can be taken to mitigate climate consequences [26,31].

This study has some limitations. As this was an ecological study, we cannot assert a causal relationship between hospitalization for cardiovascular diseases and lower temperatures. Various other well-established factors can affect the cardiovascular system, such as humidity, pollution, and diet [38], which have not been considered. Hospitalization data were collected from a national database, with data added at the time of each patient’s hospitalization. Thus, the ICD codes may have been incorrectly inputted by the attending physician. The initial objective of this research was to evaluate all cities in Brazil; however, climate data for smaller cities were incomplete or the number of hospitalizations for the evaluated ICD codes was lower than the number of days assessed. Consequently, most Brazilian cities were excluded from the analysis.

A positive aspect of our study is that it is the first Brazilian study to associate ambient temperature with hospitalization for cardiovascular diseases across more than 100 cities throughout the national territory, using updated data from the last 14 years. Furthermore, this is the only national study to compare data from cities with temperate and tropical climates.

## Conclusion

From January 2010 to June 2024, ambient temperatures below 19°C in Brazil were associated with increased hospitalizations due to cardiovascular diseases. No significant differences were observed between the tropical and temperate cities. This association may be altered by climate change, emphasizing the need for public health measures to reduce the risk of cardiovascular hospitalization.

## Supporting information

S1 Table Data of cities of south region. (DOCX)

S2 Table Data of cities of southeast region. (DOCX)

S3 Table Data of cities of central-west region. (DOCX)

S4 Table Data of cities of north region. (DOCX)

S5 Table Data of cities of northern region. (DOCX)

S1 Data The complete data on cardiovascular hospitalizations and mean temperature for all 103 cities included in the analysis during the study period. (CSV)

S2 Data Specific estimates (Relative Risk, Minimum Morbidity Temperature) for each city and region. (CSV)
