# Supplementary material for: Association between climatic variables and cardiovascular hospitalizations in Brazil: An ecological study
Source: PLOS Glob Public Health. 2026 Jul 29;6(7):e0005294. doi: 10.1371/journal.pgph.0005294 (PMC13421759; doi:10.1371/journal.pgph.0005294)
Supplement: S1 Table — (DOCX) [file pgph.0005294.s001.docx]

**Supplementary material**

**Table 1 – Data Of Cities of South Region**

| **City** | **Number of hospital admissions** | **Median temperature** | **Lower temperature mortality** | **Estimate Minimum Mortality Temperature (MMT)** | **Higher temperature mortality** | **Estimate maximum Mortality Temperature (MMT)** |
| --- | --- | --- | --- | --- | --- | --- |
| BAGE | 7584 | 18 | 12°C | 0.97 (0.88 - 1.06) | 31°C | 1.18 (0.56 - 2.49) |
| CURITIBA | 79306 | 18,3 | 24°C | 0.94 (0.87 - 1.01) | 6°C | 1.58 (0.79 - 3.17) |
| FLORIANOPOLIS | 18013 | 21,35 | 13°C | 0.96 (0.8 - 1.14) | 30°C | 1.1 (0.77 - 1.56) |
| FOZ DO IGUACU | 10269 | 22,07 | 34°C | 0.59 (0.32 - 1.09) | 28°C | 1.05 (0.93 - 1.2) |
| ITAJAI | 23729 | 20,61 | 8°C | 0.48 (0.24 - 0.97) | 26°C | 1.11 (0.95 - 1.28) |
| LAGES | 7800 | 16,66 | 27°C | 0.38 (0.12 - 1.21) | 7°C | 1.4 (1.06 - 1.86) |
| MARINGA | 23133 | 22,99 | 7°C | 0.39 (0.13 - 1.13) | 12°C | 1.28 (1.03 - 1.58) |
| PASSO FUNDO | 56499 | 18,06 | 28°C | 0.73 (0.52 - ...) | 22°C | 1.02 (0.93 - ...) |
| PELOTAS | 7210 | 18,58 | 29°C | 0.77 (0.35 - 1.68) | 22°C | 1.12 (0.94 - 1.34) |
| PORTO ALEGRE | 148337 | 20,1 | 32°C | 0.89 (0.64 - 1.22) | 6°C | 1.2 (0.87 - 1.65) |
| RIO GRANDE | 22049 | 18,78 | 25°C | 0.91 (0.8 - 1.03) | 30°C | 1.23 (0.74 - 2.04) |
| SANTA MARIA | 9688 | 19,47 | 35°C | 0.57 (0.08 - 4.24) | 28°C | 1.07 (0.9 - 1.27) |
| TRAMANDAI | 7175 | 19,98 | 22°C | 0.99 (0.94 - 1.04) | 31°C | 2.04 (0.44 - 9.4) |
| URUGUAIANA | 5324 | 19,92 | 34°C | 0.7 (0.15 - 3.4) | 5°C | 1.43 (0.82 - 2.47) |
| XANXERE | 18682 | 18,8 | 28°C | 0.87 (0.57 - 1.34) | 2°C | 1.86 (0.75 - 4.6) |
