# Supplementary material for: Association between climatic variables and cardiovascular hospitalizations in Brazil: An ecological study
Source: PLOS Glob Public Health. 2026 Jul 29;6(7):e0005294. doi: 10.1371/journal.pgph.0005294 (PMC13421759; doi:10.1371/journal.pgph.0005294)
Supplement: S2 Table — (DOCX) [file pgph.0005294.s002.docx]

**Supplementary material**

**Table 2 – Data Of Cities of Southeast region**

| **City** | **Number of hospital admissions** | **Median temperature** | **Lower temperature mortality** | **Estimate Minimum Mortality Temperature (MMT)** | **Higher temperature mortality** | **Estimate maximum Mortality Temperature (MMT)** |
| --- | --- | --- | --- | --- | --- | --- |
| BARBACENA | 20033 | 18,66 | 23°C | 0.9 (0.81 - 1) | 27°C | 1.06 (0.54 - 2.05) |
| BARRETOS | 9077 | 23,11 | 14°C | 0.96 (0.74 - 1.25) | 32°C | 1.62 (0.96 - 2.72) |
| BAURU | 22006 | 22,01 | 32°C | 0.67 (0.38 - 1.19) | 13°C | 1.14 (0.95 - 1.36) |
| BELO HORIZONTE | 118067 | 19,95 | 25°C | 0.96 (0.88 - 1.05) | 10°C | 1.18 (0.52 - 2.68) |
| CURVELO | 5788 | 23,19 | 29°C | 0.95 (0.78 - 1.15) | 34°C | 1.33 (0.28 - 6.27) |
| DIAMANTINA | 9252 | 18,62 | 23°C | 0.87 (0.75 - 1) | 13°C | 1.12 (0.94 - 1.32) |
| DIVINOPOLIS | 7102 | 22,09 | 12°C | 0.66 (0.22 - 1.98) | 30°C | 1.63 (0.73 - 3.64) |
| DUQUE DE CAXIAS | 13921 | 23,05 | 28°C | 0.97 (0.79 - 1.18) | 14°C | 1.47 (0.55 - 3.92) |
| FORMIGA | 5305 | 21,71 | 29°C | 0.81 (0.56 - 1.18) | 12°C | 1.25 (0.59 - 2.67) |
| FRANCA | 15455 | 21,73 | 10°C | 0.86 (0.3 - 2.45) | 14°C | 1.17 (0.89 - 1.53) |
| GOVERNADOR VALADARES | 19954 | 24,44 | 35°C | 0.28 (0.05 - 1.55) | 31°C | 1.09 (0.86 - 1.39) |
| ITAPEVA | 6524 | 19,98 | 6°C | 0.31 (0.04 - 2.3) | 30°C | 1.47 (0.76 - 2.86) |
| JALES | 6069 | 24,74 | 10°C | 0.75 (0.24 - 2.34) | 16°C | 1.02 (0.78 - 1.34) |
| JUIZ DE FORA | 44169 | 19,37 | 29°C | 0.79 (0.36 - 1.73) | 9°C | 1.57 (0.65 - 3.75) |
| LINHARES | 14371 | 24,36 | 28°C | 0.96 (0.83 - 1.12) | 18°C | 1.28 (0.75 - 2.17) |
| MACAE | 7505 | 23,55 | 33°C | 0.7 (0.34 - 1.42) | 17°C | 1.16 (0.76 - 1.76) |
| MARILIA | 9117 | 22,79 | 18°C | 0.99 (0.93 - 1.05) | 33°C | 2.24 (1.13 - 4.46) |
| MONTES CLAROS | 46167 | 24,03 | 28°C | 0.96 (0.87 - 1.06) | 16°C | 1.31 (0.55 - 3.13) |
| MURIAE | 23236 | 23,28 | 29°C | 0.92 (0.79 - 1.06) | 15°C | 1.88 (1.11 - 3.19) |
| NITEROI | 5578 | 24,83 | 17°C | 0.61 (0.27 - 1.37) | 33°C | 1.52 (0.6 - 3.84) |
| NOVA FRIBURGO | 14295 | 17,08 | 8°C | 0.47 (0.24 - 0.91) | 27°C | 2.03 (0.79 - 5.22) |
| OURINHOS | 6680 | 22,24 | 8°C | 0.17 (0.04 - 0.67) | 13°C | 1.28 (0.97 - 1.7) |
| PASSOS | 13017 | 21,58 | 33°C | 0.5 (0.09 - 2.58) | 11°C | 1.04 (0.56 - 1.93) |
| PATOS DE MINAS | 2903 | 22,06 | 30°C | 0.69 (0.33 - 1.48) | 23°C | 1 (0.98 - 1.03) |
| PIRACICABA | 16493 | 22,01 | 10°C | 0.56 (0.22 - 1.39) | 31°C | 1.38 (0.75 - 2.54) |
| PRESIDENTE PRUDENTE | 27239 | 23,97 | 34°C | 0.58 (0.34 - 0.97) | 14°C | 1.15 (0.94 - 1.41) |
| RIO DE JANEIRO | 69119 | 23,23 | 28°C | 0.92 (0.81 - 1.05) | 15°C | 1.13 (0.63 - 2.04) |
| SETE LAGOAS | 9060 | 22 | 12°C | 0.39 (0.15 - 1) | 17°C | 1.42 (1.12 - 1.8) |
| SOROCABA | 34631 | 20,91 | 25°C | 0.95 (0.88 - 1.02) | 8°C | 1.97 (0.7 - 5.56) |
| SAO CARLOS | 16000 | 21,19 | 30°C | 0.67 (0.41 - 1.09) | 12°C | 1.3 (1.03 - 1.65) |
| SAO GONCALO | 22210 | 27,54 | 33°C | 0.13 (0.01 - 2.1) | 22°C | 1.81 (0.26-12.42) |
| SAO JOAO DEL REI | 6048 | 19,82 | 14°C | 0.92 (0.75 - 1.12) | 11°C | 1.34 (0.68 - 2.64) |
| SAO MATEUS | 6779 | 24 | 28°C | 0.91 (0.74 - 1.11) | 32°C | 3.15 (0.39-25.67) |
| SAO PAULO | 450734 | 20,56 | 24°C | 0.98 (0.95 - 1.01) | 8°C | 1.11 (0.63 - 1.95) |
| SÃO SEBASTIAO DO PARAISO | 5776 | 21,21 | 9ºC | 0.58 (0.07 - 4.58) | 14°C | 1.2 (0.84 - 1.72) |
| TAUBATE | 15,956 | 21.10 | 20°C | 1 (1 - 1) | 10°C | 2.02 (0.75 - 5.44) |
| TEOFILO OTONI | 7,194 | 23.30 | 16°C | 0.71 (0.3 - 1.66) | 31°C | 1.24 (0.63 - 2.42) |
| TUPA | 3,159 | 24.02 | 10°C | 0.47 (0.09 - 2.33) | 15°C | 1.25 (0.74 - 2.11) |
| UBERABA | 10,922 | 22.79 | 34°C | 0.42 (0.15 - 1.2) | 8°C | 2.67 (0.26-27.55) |
| UBERLANDIA | 31,690 | 23.53 | 16°C | 0.95 (0.74 - 1.22) | 11°C | 1.11 (0.36 - 3.42) |
| VARGINHA | 17,743 | 20.40 | 25°C | 0.97 (0.86 - 1.08) | 10°C | 1.55 (0.55 - 4.39) |
| VILA VELHA | 13,986 | 23.92 | 32°C | 0.34 (0.09 - 1.23) | 29°C | 1.12 (0.81 - 1.56) |
| VITORIA | 22,898 | 24.51 | 18°C | 0.91 (0.65 - 1.28) | 32°C | 1.23 (0.85 - 1.79) |
| VOTUPORANGA | 9,619 | 24.50 | 11°C | 0.66 (0.16 - 2.71) | 33°C | 1.09 (0.67 - 1.76) |
