# Supplementary material for: Association between climatic variables and cardiovascular hospitalizations in Brazil: An ecological study
Source: PLOS Glob Public Health. 2026 Jul 29;6(7):e0005294. doi: 10.1371/journal.pgph.0005294 (PMC13421759; doi:10.1371/journal.pgph.0005294)
Supplement: S3 Table — (DOCX) [file pgph.0005294.s003.docx]

**Supplementary material**

**Table 3 – Data of Cities of Central-west region**

| **City** | **Number of hospital admissions** | **Median temperature** | **Lower temperature mortality** | **Estimate Minimum Mortality Temperature (MMT)** | **Higher temperature mortality** | **Estimate maximum Mortality Temperature (MMT)** |
| --- | --- | --- | --- | --- | --- | --- |
| BRASILIA | 58276 | 21,56 | 29°C | 0.68 (0.4 - 1.13) | 12°C | 1.31 (0.55 - 3.1) |
| CAMPO GRANDE | 46272 | 24,12 | 28°C | 0.91 (0.84 - 0.99) | 8°C | 1.93 (1.03 - 3.61) |
| CATALAO | 6853 | 23,31 | 11°C | 0.23 (0.04 - 1.31) | 16°C | 1.47 (0.93 - 2.31) |
| CUIABA | 22591 | 27,19 | 12°C | 0.5 (0.22 - 1.14) | 17°C | 1.28 (0.95 - 1.73) |
| DOURADOS | 10961 | 23,38 | 28°C | 0.93 (0.82 - 1.07) | 34°C | 1.76 (0.99 - 3.13) |
| GOIANIA | 118238 | 23,85 | 12°C | 0.38 (0.09 - 1.63) | 16°C | 1.39 (1.01 - 1.9) |
| RIO BRANCO | 7884 | 25,72 | 15°C | 0.39 (0.19 - 0.83) | 29°C | 1.13 (0.97 - 1.32) |
| RONDONOPOLIS | 11366 | 25,7 | 34°C | 0.89 (0.71 - 1.12) | 15°C | 1.57 (1.21 - 2.04) |
