# Supplementary material for: Association between climatic variables and cardiovascular hospitalizations in Brazil: An ecological study
Source: PLOS Glob Public Health. 2026 Jul 29;6(7):e0005294. doi: 10.1371/journal.pgph.0005294 (PMC13421759; doi:10.1371/journal.pgph.0005294)
Supplement: S4 Table — (DOCX) [file pgph.0005294.s004.docx]

**Supplementary material**

**Table 4 – Data Of Cities of North Region**

| **City** | **Number of hospital admissions** | **Median temperature** | **Lower temperature mortality** | **Estimate Minimum Mortality Temperature (MMT)** | **Higher temperature mortality** | **Estimate maximum Mortality Temperature (MMT)** |
| --- | --- | --- | --- | --- | --- | --- |
| BOA VISTA | 8916 | 27,57 | 35°C | 0.18 (0.03 - 1.01) | 26°C | 1.07 (0.99 - 1.15) |
| CACOAL | 4421 | 26,68 | 37°C | 0.61 (0.22 - 1.71) | 13°C | 4.88(0.22-10.68) |
| MACAPA | 7908 | 27,37 | 25°C | 0.91 (0.71 - 1.17) | 33°C | 1.37 (0.27 - 7) |
| MANAUS | 47130 | 27,78 | 33°C | 0.81 (0.63 - 1.05) | 25°C | 1.07 (0.95 - 1.2) |
| PALMAS | 14606 | 27,55 | 22°C | 0.57 (0.26 - 1.24) | 36°C | 1.04 (0.17 - 6.51) |
| PORTO VELHO | 11097 | 26,67 | 22°C | 0.87 (0.65 - 1.16) | 19°C | 2.2 (0.96 - 5.09) |
| RIO BRANCO | 7884 | 25,72 | 15°C | 0.39 (0.19 - 0.83) | 29°C | 1.13 (0.97 - 1.32) |
| SANTAREM | 6354 | 26,7 | 30°C | 0.78 (0.49 - 1.26) | 29°C | 1.14 (0.93 - 1.4) |
