# Supplementary material for: Association between climatic variables and cardiovascular hospitalizations in Brazil: An ecological study
Source: PLOS Glob Public Health. 2026 Jul 29;6(7):e0005294. doi: 10.1371/journal.pgph.0005294 (PMC13421759; doi:10.1371/journal.pgph.0005294)
Supplement: S5 Table — (DOCX) [file pgph.0005294.s005.docx]

| **City** | **Number of hospital admissions** | **Median temperature** | **Lower temperature mortality** | **Estimate Minimum Mortality Temperature (MMT)** | **Higher temperature mortality** | **Estimate maximum Mortality Temperature (MMT)** |
| --- | --- | --- | --- | --- | --- | --- |
| ARAPIRACA | 9944 | 24,88 | 23°C | 0.78 (0.66 - 0.92) | 31°C | 1.26 (0.56 - 2.82) |
| BARBALHA | 7307 | 26,36 | 19°C | 0.54 (0.06 - 4.9) | 22°C | 1.24 (0.87 - 1.77) |
| BARREIRAS | 6401 | 25,81 | 30°C | 0.98 (0.85 - 1.14) | 18°C | 1.43 (0.13 - 15.4) |
| BOA VISTA | 8916 | 27,57 | 35°C | 0.18 (0.03 - 1.01) | 26°C | 1.07 (0.99 - 1.15) |
| CAMPINA GRANDE | 17872 | 23,81 | 29°C | 0.78 (0.29 - 2.14) | 28°C | 1.1 (0.69 - 1.76) |
| CARUARU | 18149 | 22,51 | 27°C | 0.76 (0.53 - 1.08) | 19°C | 1.21 (1.04 - 1.42) |
| FEIRA DE SANTANA | 13732 | 24,78 | 33°C | 0.52 (0.27 - 1) | 19°C | 1.44 (0.82 - 2.54) |
| FORTALEZA | 132794 | 27,45 | 30°C | 0.97 (0.9 - 1.05) | 32°C | 1.08 (0.82 - 1.44) |
| ILHEUS | 6865 | 23,71 | 29°C | 0.27 (0.06 - 1.33) | 19°C | 1.14 (0.65 - 2.03) |

**Supplementary material**

**Table 5 – Data Of Cities of Northern Region**
