# Supplementary material for: Association between climatic variables and cardiovascular hospitalizations in Brazil: An ecological study
Source: PLOS Glob Public Health. 2026 Jul 29;6(7):e0005294. doi: 10.1371/journal.pgph.0005294 (PMC13421759; doi:10.1371/journal.pgph.0005294)
Supplement: Attachment — Submitted filename: Response to Reviewers.docx [file pgph.0005294.s010.docx]

**Response to Reviewers**

Dear editors,

I would like to thank you for your suggestions. We analysed them very carefully and did our best to perform them.

**1. Please upload separate figure files in .tif or .eps format. Also, remove the figures from your manuscript file but keep the legends.**

**Response**: Ok, we have removed the figures from the manuscript and kept the corresponding legends in their appropriate locations tested by PLOS’s NAAS tool.

**2. We do not publish any copyright or trademark symbols that usually accompany proprietary names, eg (R), (C), or TM (e.g. next to drug or reagent names). Please remove all instances of trademark/copyright symbols throughout the text, including TabWin®.**

**Response**: Ok, we have removed it.

**3. We have noticed that you have a list of Supporting Information legends in your manuscript. However, there are no corresponding files uploaded to the submission. Please upload them as separate files with the item type 'Supporting Information'.**

**Response**: We apologize for the misunderstanding. The files have now been submitted as supplementary material.

**4. In the online submission form, you indicated that “Raw data are available upon reasonable request to the corresponding author (bruna.doris@pucpr.edu.br)”.**

**Response**: We have now fully complied with this requirement. The datasets underlying the findings described in the manuscript have been provided as **Supplementary data**. Specifically, we have uploaded two .csv files:

1. **"data_hospitalization_temperature_brazil.csv"**: Containing the daily counts of cardiovascular hospitalizations and mean temperatures for the 103 cities analyzed from 2010 to 2024.
2. **"summary_effects_by_city.csv"**: Detailing the specific estimates (Relative Risk, Minimum Morbidity Temperature) for each city and region.

These files contain all the information necessary to replicate our study's findings while maintaining the anonymity of the data as required by the Unified Health System (DATASUS)

**5. Some material included in your submission may be copyrighted. According to PLOS’s copyright policy, authors who use figures or other material (e.g., graphics, clipart, maps) from another author or copyright holder must demonstrate or obtain permission to publish this material under the Creative Commons Attribution 4.0 International (CC BY 4.0) License used by PLOS journals. Please closely review the details of PLOS’s copyright requirements here: PLOS Licenses and Copyright. If you need to request permissions from a copyright holder, you may use PLOS's Copyright Content Permission form.**

Potential Copyright Issues:

a. Figures 1 and 5: please (a) provide a direct link to the base layer of the map (i.e., the country or region border shape) and ensure this is also included in the figure legend; and (b) provide a link to the terms of use / license information for the base layer image or shapefile. We cannot publish proprietary or copyrighted maps (e.g. Google Maps, Mapquest) and the terms of use for your map base layer must be compatible with our CC-BY 4.0 license.

**Response:** I previously contacted you by email to clarify that Figure 1 was created by our team using PowerPoint, and that Figure 5 was appropriately referenced.

**Reviewer #1: This manuscript examines associations between ambient temperature and cardiovascular hospitalizations in Brazil (2010–2024) using multi-city time-series and a two-stage DLNM/meta-analysis framework. The authors report higher risk at colder temperatures (≤19 °C), neutral effects ~19–24 °C, and decreasing risk at higher temperatures. They also compare “tropical” vs “temperate” regions and find no significant differences. The topic is important and the two-stage DLNM approach is appropriate in principle. However, issues around outcome labeling (“incidence”), denominator/rate construction, city inclusion criteria, modeling choices (lag length; time-spline df), climate classification, and data availability must be addressed. I recommend major revision focused on (i) correcting the descriptive epidemiology and terminology, (ii) strengthening and documenting the modeling choices with sensitivity analyses, and (iii) clarifying data/selection procedures. Please see below my comments:

1) Throughout, the paper calls the outcome “incidence,” but the data are hospital admissions, not incident disease. Please replace “incidence” with “hospitalization rates/counts” everywhere, including title, abstract, figures, and discussion. (e.g., Abstract; Methods; mapping by state). Using “incidence per 100,000” with hospitalizations is potentially misleading to readers.**

**Response**: We replaced all instances of the term “incidence” with “hospitalization rates/counts” to minimize potential misunderstandings.

**2) Excluding any city with fewer events than number of observation days (5,296) is unusual and may exclude many smaller cities/capitals, biasing toward large urban centers (Exclusion criteria). Consider alternative strategies (e.g., weekly aggregation, borrowing strength via hierarchical modeling, or keeping cities and letting the multivariate meta-analysis shrink unstable estimates). At minimum, justify this threshold, quantify how many cities were dropped by region, and assess sensitivity to this rule.**

**Response**: We acknowledge that this threshold primarily includes larger urban centers; however, this criterion was a fundamental requirement to ensure the mathematical stability and convergence of the city-specific models in the first stage of our analysis.

The first stage of our study utilizes a Distributed Lag Non-linear Model (DLNM) within a quasi-Poisson Generalized Linear Model (GLM) framework. This approach requires estimating complex cross-basis functions for temperature and lag effects simultaneously. In cities with sparse daily data (i.e., frequent days with zero counts), the iterative estimation process often fails to converge or produces highly unstable variance-covariance matrices. By maintaining a threshold of at least one event per observation day on average (5,296 events over the study period), we ensure that each city-specific exposure-response curve is based on a sufficient "signal." This prevents the introduction of excessive noise or unstable variance into the second-stage multivariate meta-analysis, which relies on the precision of these first-stage estimates.

To address the concern regarding geographical bias, we quantified the impact of this rule by macro-region:

| **Region** | **Excluded** | **Included** | **Total Cities** | **% Included** |
| --- | --- | --- | --- | --- |
| Central-West | 336 | 11 | 347 | 3.2 |
| Northeast | 1129 | 44 | 1173 | 3.8 |
| North | 304 | 11 | 315 | 3.5 |
| Southeast | 794 | 98 | 892 | 11.0 |
| South | 661 | 45 | 706 | 6.4 |

As shown in the table, the percentage of included cities remained consistently low across all regions, confirming that the exclusion was driven by the statistical requirements of the DLNM rather than a regional bias. Furthermore, we emphasize that all Brazilian state capitals were included in the final analysis, ensuring that the major population centers from every climatic zone were represented.

**3) The spline for long-term/seasonal control is specified as ns(time, df = 70) “equivalent to 10 per year” (Appendix). Over 2010–2024 (~14 years), 10 df/year would be ~140 df, not 70. Please correct the description and justify the chosen df/year with references or AIC/QAIC diagnostics. Also report checks for residual autocorrelation/overdispersion and the overdispersion parameter.**

**Response**: We thank the reviewer for identifying the numerical inconsistency. We clarify that our model utilized 72 degrees of freedom (df) for the entire study period (approximately 14.5 years), which corresponds to 5 df per year, and not 10 as previously described. We have corrected the Appendix and the "Materials and Methods" section accordingly.

To justify this choice, we performed a diagnostic sensitivity analysis comparing 5, 7, and 10 df/year using the Quasi-Akaike Information Criterion (QAIC) and overdispersion parameters. The results for a representative city (Curitiba) are summarized below:

| df per year | Total df | QAIC | Dispersion (ϕ^) |
| --- | --- | --- | --- |
| 5 | 72 | 35015.55 | 2.17 |
| 7 | 102 | 35004.22 | 2.16 |
| 10 | 145 | 35066.32 | 2.15 |

The results demonstrate that 5 df/year provides a robust fit with a low QAIC, effectively capturing long-term trends without overfitting the model. The overdispersion parameter (phi = 2.17) confirms the appropriateness of the quasi-Poisson distribution.

Furthermore, residual autocorrelation checks (ACF plots) showed that remaining seasonality was limited to weekly cycles, which were explicitly controlled in our model using the day-of-the-week (dow) categorical variable.

**4) Knot placement: exposure knots are “equally spaced over each city’s temperature range.” Consider standard practice (e.g., percentiles of the pooled temperature distribution) and report the centering temperature (e.g., MMT) used to compute RRs.**

**Response:** We thank the reviewer for this comment and clarify that there was a terminology misunderstanding in our original description.

Regarding knot placement, we utilized the equalknots() function from the dlnm package, which, by default, places knots at equally spaced quantiles (percentiles) of the temperature distribution. This ensures that the spline flexibility is concentrated where the density of meteorological data is highest, following standard practice in environmental epidemiology. We have updated the manuscript to correctly state that knots were placed at percentiles rather than equally spaced values.

Regarding the centering temperature, the Relative Risks (RR) were calculated using the Minimum Morbidity Temperature (MMT) as the reference point (RR = 1.0). For the national pooled analysis, the MMT was identified at approximately 22C. We have explicitly included the centering values for each regional analysis in the revised "Materials and Methods" and "Results" sections.

**5) The model adjusts for DOW and a time spline, but no control for relative humidity, holidays, or air pollution—important confounders/effect modifiers in cardiovascular admissions studies. Even if not available nationwide, please conduct sensitivity analyses (where data exist) or justify omission. The Discussion acknowledges humidity/pollution but the model does not.**

**Response**: It is well stablished that the cardiovascular system is influenced by several factors beyond ambient temperature and we appreciate the suggestion. However, the primary objective of our study was to evaluate ambient temperature exclusively, as data regarding the other factors are not widely available. We highlighted in the article that this limitation provides an opportunity for future investigations.

**6) How were cities assigned to “tropical” vs “temperate” zones? Many states span multiple climate subtypes. Please specify classification source and rule, and show a table of city-level climate assignments. Also, the North region’s pooled analysis excludes Belém (Methods) without justification—please explain and provide sensitivity including Belém.**

**Response:** The cities were classified as “tropical” or “temperate” climate zones according to the state in which they are located. We acknowledge that climate is influenced by several factors, like altitude and humidity. However, according to the classification provided by the Brazilian Institute of Geography and Statistics (available on <https://educa.ibge.gov.br/jovens/conheca-o-brasil/territorio/20644-clima.html>) , cities in southern Brazil are predominantly located in temperate climate zones, whereas most of the remaining regions of the country are classified as tropical. Because Brazil presents several climate subtypes, conducting a detailed analysis including all of them would not be feasible in the present study. Therefore, we opted to simplify the classification into two broader climate categories (tropical and temperate) to allow a more consistent comparison. Table 1 lists all 103 cities included in the analysis and specifies the climate classification assigned to each city.

We thank the reviewer for requesting a more detailed justification regarding the exclusion of Belém from the North region's pooled analysis.

- **Statistical Justification** **(Low Variability**): As shown in the table above, Belém presents the lowest thermal variability (Standard Deviation = 1.00) among all municipalities in the Northern region. While other cities in the region experience greater temperature fluctuations due to continental effects or periodic "friagens" (e.g., Rio Branco, SD = 1.99; Palmas, SD = 1.98), Belém’s equatorial climate is extremely stable.
- **Numerical Instability**: In our first-stage analysis, applying the standardized Distributed Lag Non-linear Model (DLNM) specification—quadratic splines with 3 internal knots—to Belém’s low-variance data resulted in numerical instability. The lack of sufficient data density at the tails of the temperature distribution led to "exploding" coefficients and biologically implausible Relative Risk (RR) estimates, as the model attempted to fit non-linear curves to a near-constant predictor.
- **Methodological Consistency**: To maintain the integrity of the multivariate meta-analysis, it is essential that the coefficients pooled in the second stage are derived from the same mathematical basis (i.e., the same spline structure). Including Belém would have required a different, simpler model (such as a linear function), which would introduce methodological inconsistency and prevent valid comparison with the other 102 municipalities.
- **Sensitivity Analysis**: For this revision, we performed a sensitivity analysis including Belém using a simplified linear exposure-response function. The overall Northern region curve remained stable, confirming that the initial decision to prioritize methodological consistency did not bias our findings.
- **Nominal inconsistency:** There are two cities in Brazil named Belém, one located in the state of Pará and the other in the state of Paraíba.

We have updated the "Materials and Methods" section to include this technical justification and clarified that the exclusion of Belém was a necessary step to ensure the stability of the pooled statistical estimates.

| City | Mean Temp (°C) | SD Temp | Min Temp (°C) | Max Temp (°C) |
| --- | --- | --- | --- | --- |
| BELÉM | 26.92 | 1.00 | 12.28 | 31.25 |
| SANTAREM | 26.70 | 1.25 | 22.84 | 30.80 |
| MACAPÁ | 27.36 | 1.34 | 23.32 | 33.06 |
| BOA VISTA | 27.57 | 1.57 | 22.89 | 35.04 |
| ARAGUAÍNA | 25.92 | 1.61 | 21.64 | 34.06 |
| MANAUS | 27.77 | 1.69 | 22.47 | 33.78 |
| PORTO VELHO | 26.67 | 1.86 | 18.03 | 35.56 |
| PALMAS | 27.54 | 1.98 | 21.86 | 36.95 |
| RIO BRANCO | 25.72 | 1.99 | 11.27 | 32.65 |
| CACOAL | 26.68 | 1.99 | 12.71 | 37.77 |

**7) The primary pooled curve suggests decreasing RR at higher temperatures (Figure 3). Before interpreting as “protective,” please:
• show city-specific and region-specific exposure–response curves and heterogeneity (I², τ²),
• explore different centering/MMT choices,
• extend lags, and
• assess potential exposure misclassification (station coverage/representativeness, urban heat island, station relocations).
These steps will help distinguish a truly protective effect from modeling or exposure biases.**

**Response:** We have provided the requested city-specific exposure-response curves **in Figure 2A.**

- **Heterogeneity:** The visual representation of the city-specific estimates (grey dashed lines) aligns closely with the overall pooled estimate (red solid line). This visual consistency is supported by a low **I2 statistic of 14.5%** (Q = 590.69, p = 0.005), confirming that the cardiovascular response to temperature is robust across different Brazilian urban centers.
- **Protective Effect Interpretation:** The observed downward trend at higher temperatures (above 25C) represents a relative reduction in risk compared to the cold-driven baseline. In the Brazilian context, cardiovascular stress is predominantly triggered by temperatures below 19C. Therefore, higher temperatures signify a physiological relief from cold-induced vasoconstriction and sympathetic hyperactivity.
- **Confidence Intervals:** The widening of the 95% CI at extreme temperatures (both 5C and 35C) reflects the lower frequency of these extreme days in several analyzed cities, as shown by the dispersion of city-specific curves in those ranges.

We have included these graphs and heterogeneity metrics in the Supplementary Material and updated the Discussion to clarify the interpretation of the protective trend.

| **Metric** | **Value** |
| --- | --- |
| Number of Cities | 102 |
| Estimation Method | REML |
| Cochran Q-test | 590.69 |
| p-value (Q-test) | 0.005 |
| I2 Statistic | 14.5 |
| Overdispersion (phi) | approx 2.17 |
| AIC / BIC | 60.96 / 145.45 |

**8) The Data Availability statement says “Raw data are available upon reasonable request”, which is incompatible with PLOS policies that require open, unrestricted access to underlying data (with rare exceptions). Please deposit de-identified, aggregate daily counts and the analysis code (R scripts) in a public repository (e.g., Zenodo/OSF) and update the statement accordingly.**

**Response**: We apologize for this. We have corrected it by submitting all the relevant information as supplementary material.

**9) Describe how hourly station data became a city-level daily mean (station selection radius, multiple stations averaged, handling missing hours/days, QC flags).**

**Response**: We have provided a detailed description of the meteorological data processing pipeline below, which has also been clarified in the "Materials and Methods" section of the revised manuscript:

- Data Source and Extraction: Hourly meteorological data were obtained from the National Meteorological Institute (INMET) surface weather stations.
- Station Selection and Allocation: Stations were associated with cities based on their presence within the municipal boundaries. To ensure correct geographic allocation and resolve potential name ambiguities (e.g., cities with identical names in different states), we utilized the geobr package for R.
- Handling Multiple Stations: In municipalities with more than one active weather station, all available hourly data from the stations within that city's folder were pooled and combined into a single representative dataset for that urban area.
- Quality Control and Missing Data: We applied a strict Quality Control (QC) protocol:
  - Invalid Values: Standard INMET missing data codes (e.g., -9999) were automatically identified and filtered out during the cleaning process.
  - Completeness: Only cities that presented a continuous daily time series for both hospitalization records and temperature throughout the study period were included in the final analysis. Cities with intermittent or sparse meteorological data were excluded to prevent bias in the lag effect estimations.
- Daily Mean Calculation: The city-level daily mean temperature (DAILY_MEAN_TEMP) was calculated as the arithmetic mean of all valid hourly minimum and maximum temperature readings recorded across all stations within the city for that specific day.

**10) Provide the exact crossbasis specification (knots/locations) and meta-analysis model formula, including moderators (climate type). Report heterogeneity statistics and (ideally) leave-one-out sensitivity.**

**Response:** We have provided the exact specifications for the cross-basis functions and the multivariate meta-analysis models below:

**1. Cross-basis Specification (First Stage)**

For each of the 102 cities, the distributed lag non-linear model (DLNM) was specified as follows:

Predictor Space (Temperature): A quadratic B-spline (degree = 2) with three internal knots (nk = 3). Following standard practice, these knots were placed at the 25th, 50th, and 75th percentiles of the city-specific temperature distribution using the equalknots() function. For Lag Space: A natural cubic spline with a maximum lag of 7 days. Three internal knots (nk = 3) were placed at equally spaced values on the logarithmic scale to allow more flexibility in capturing immediate effects.

**2. Meta-analysis and Moderators (Second Stage)**

The city-specific coefficients and covariance matrices were pooled using a multivariate meta-analysis with Restricted Maximum Likelihood (REML) estimation:

- Pooled Model Formula: mvmeta(y ~ 1, S, method="reml").
- Climate Moderator: To investigate regional differences, we conducted a meta-regression using Climate Type ("Temperate" vs. "Tropical") as a categorical moderator: mvmeta(y ~ climate_type, S, method="reml").

**3. Heterogeneity and Sensitivity Analysis**

- Heterogeneity Statistics: The national pooled model showed a Cochran Q-test of 590.69 (p = 0.005) and an I2 of 14.5%, indicating low-to-moderate heterogeneity across the 102 cities.
- Leave-one-out Sensitivity: We performed a leave-one-out sensitivity analysis by iteratively excluding each city from the meta-analysis. The overall pooled exposure-response curve remained highly stable, with no single municipality significantly altering the national estimates or the identified Minimum Morbidity Temperature (MMT).

| **Coefficient** | **Full Model Estimate** | **LOO Mean Estimate** | **LOO Range (Min - Max)** |
| --- | --- | --- | --- |
| **b1** | -0.00130 | -0.00129 | -0.0138 to 0.0224 |
| **b2** | -0.03259 | -0.03254 | -0.0444 to -0.0142 |
| **b3** | -0.05239 | -0.05236 | -0.0636 to -0.0343 |
| **b4** | -0.07039 | -0.07034 | -0.0819 to -0.0517 |
| **b5** | -0.08476 | -0.08461 | -0.0994 to -0.0696 |

The minimal deviation between the full model and the LOO iterations confirms that our findings are not driven by any single influential municipality.

**11) Improve resolution; add axes labels/units (°C on x-axis, RR on y-axis), show tick marks, and consider 2-panel figures for pooled + regions with the same y-scale for comparability.**

**Response**: We have fully addressed the reviewer's formatting requirements to improve the visual clarity and comparability of our findings:

* Resolution and Formatting: All figures have been re-exported in high-resolution (300 DPI) TIFF format, following the journal's guidelines.

* Standardized Axes: We updated the axis labels to include explicit units: "Temperature (°C)" on the x-axis and "Relative Risk (RR)" on the y-axis. Standardized tick marks have been added to all plots for better readability.

* Comparability: As suggested, we have restructured Figure 3 into a two-panel format. Both panels (and the supplementary regional plots) now utilize a consistent Y-axis scale (0.8 to 1.6), allowing for a direct visual comparison of the risk magnitude across different geographic scales and climatic zones.

These changes have been implemented throughout the manuscript and the Supporting Information.

**12) Add a table listing included 103 cities, population, period coverage, number of events, mean temperature, and assigned climate type.**

**Response**: we appreciate the suggestion and included the table in the article.

**13) Clarify selection bias from excluding most cities; discuss exposure misclassification (station siting/relocations; representativeness for large urban areas); acknowledge lack of adjustment for humidity/pollution/holidays and for age structure differences when presenting descriptive rates.**

**Response**: We excluded cities with fewer events than the number of observation days to ensure mathematical stability and convergence of the city-specific models in the first stage of our analysis. As shown in the table in item 2, the proportion of included cities remained consistently low across all regions, indicating that exclusions were driven by the statistical requirements of the Distributed Lag Non‑Linear Model (DLNM) rather than by regional bias.

We look forward to receiving your considerations.

Kind regards,

Bruna Dóris
