# Supplementary material for: Association between climatic variables and cardiovascular hospitalizations in Brazil: An ecological study
Source: PLOS Glob Public Health. 2026 Jul 29;6(7):e0005294. doi: 10.1371/journal.pgph.0005294 (PMC13421759; doi:10.1371/journal.pgph.0005294)
Supplement: Peer review history

# Peer review: Decision Letter 0

**DOI:** 10.1371/journal.pgph.0005294.r001

**Authors:** Collins Asweto

8 Feb 2026

PGPH-D-25-02810

ASSOCIATION BETWEEN CLIMATIC VARIABLES AND CARDIOVASCULAR HOSPITALIZATIONS IN BRAZIL: AN ECOLOGICAL STUDY

PLOS Global Public Health

Dear Doris,

Thank you for submitting your manuscript to PLOS Global Public Health. After careful consideration, we feel that it has merit but does not fully meet PLOS Global Public Health’s publication criteria as it currently stands. Therefore, we invite you to submit a revised version of the manuscript that addresses the points raised during the review process.

Please submit your revised manuscript by8th April 2026. If you will need more time than this to complete your revisions, please reply to this message or contact the journal office at globalpubhealth@plos.org. When you're ready to submit your revision, log on to https://www.editorialmanager.com/pgph/ and select the 'Submissions Needing Revision' folder to locate your manuscript file.

Please include the following items when submitting your revised manuscript:

- A letter that responds to each point raised by the editor and reviewer(s). You should upload this letter as a separate file labeled 'Response to Reviewers'.
- A marked-up copy of your manuscript that highlights changes made to the original version. You should upload this as a separate file labeled 'Revised Manuscript with Track Changes'.
- An unmarked version of your revised paper without tracked changes. You should upload this as a separate file labeled 'Manuscript'.

Guidelines for resubmitting your figure files are available below the reviewer comments at the end of this letter.

We look forward to receiving your revised manuscript.

Kind regards,

Collins Otieno Asweto, PhD

Academic Editor

PLOS Global Public Health

Journal Requirements:

1. Please upload separate figure files in .tif or .eps format. Also, remove the figures from your manuscript file but keep the legends.

For more information about figure files please see our guidelines:

https://journals.plos.org/globalpublichealth/s/figures

https://journals.plos.org/globalpublichealth/s/figures#loc-file-requirements

2. We do not publish any copyright or trademark symbols that usually accompany proprietary names, eg (R), (C), or TM  (e.g. next to drug or reagent names). Please remove all instances of trademark/copyright symbols throughout the text, including TabWin®.

3. We have noticed that you have a list of Supporting Information legends in your manuscript. However, there are no corresponding files uploaded to the submission. Please upload them as separate files with the item type 'Supporting Information'.

4. In the online submission form, you indicated that “Raw data are available upon reasonable request to the corresponding author (bruna.doris@pucpr.edu.br)”.

All PLOS journals now require all data underlying the findings described in their manuscript to be freely available to other researchers, either

1. In a public repository,

2. Within the manuscript itself, or

3. Uploaded as supplementary information.

This policy applies to all data except where public deposition would breach compliance with the protocol approved by your research ethics board. If your data cannot be made publicly available for ethical or legal reasons (e.g., public availability would compromise patient privacy), please explain your reasons by return email and your exemption request will be escalated to the editor for approval. Your exemption request will be handled independently and will not hold up the peer review process, but will need to be resolved should your manuscript be accepted for publication. One of the Editorial team will then be in touch if there are any issues.

5. Some material included in your submission may be copyrighted. According to PLOS’s copyright policy, authors who use figures or other material (e.g., graphics, clipart, maps) from another author or copyright holder must demonstrate or obtain permission to publish this material under the Creative Commons Attribution 4.0 International (CC BY 4.0) License used by PLOS journals. Please closely review the details of PLOS’s copyright requirements here: PLOS Licenses and Copyright. If you need to request permissions from a copyright holder, you may use PLOS's Copyright Content Permission form.

Please respond directly to this email or email the journal office and provide any known details concerning your material's license terms and permissions required for reuse, even if you have not yet obtained copyright permissions or are unsure of your material's copyright compatibility.

Potential Copyright Issues:

a. Figures 1 and 5: please (a) provide a direct link to the base layer of the map (i.e., the country or region border shape) and ensure this is also included in the figure legend; and (b) provide a link to the terms of use / license information for the base layer image or shapefile. We cannot publish proprietary or copyrighted maps (e.g. Google Maps, Mapquest) and the terms of use for your map base layer must be compatible with our CC-BY 4.0 license.

Note: if you created the map in a software program like R or ArcGIS, please locate and indicate the source of the basemap shapefile onto which data has been plotted.

If your map was obtained from a copyrighted source please amend the figure so that the base map used is from an openly available source. Alternatively, please provide explicit written permission from the copyright holder granting you the right to publish the material under our CC-BY 4.0 license.

Please note that the following CC BY licenses are compatible with PLOS license: CC BY 4.0, CC BY 2.0 and CC BY 3.0, meanwhile such licenses as CC BY-ND 3.0 and others are not compatible due to additional restrictions.

If you are unsure whether you can use a map or not, please do reach out and we will be able to help you. The following websites are good examples of where you can source open access or public domain maps:

* U.S. Geological Survey (USGS) - All maps are in the public domain. (http://www.usgs.gov)

* PlaniGlobe - All maps are published under a Creative Commons license so please cite “PlaniGlobe, http://www.planiglobe.com, CC BY 2.0” in the image credit after the caption. (http://www.planiglobe.com/?lang=enl)

* Natural Earth - All maps are public domain. (http://www.naturalearthdata.com/about/terms-of-use/)

If the reviewer comments include a recommendation to cite specific previously published works, please review and evaluate these publications to determine whether they are relevant and should be cited. There is no requirement to cite these works unless the editor has indicated otherwise.

Additional Editor Comments (if provided):

[Note: HTML markup is below. Please do not edit.]

Reviewers' comments:

Reviewer's Responses to Questions

**Comments to the Author**

1. Does this manuscript meet PLOS Global Public Health’s publication criteria? Is the manuscript technically sound, and do the data support the conclusions? The manuscript must describe methodologically and ethically rigorous research with conclusions that are appropriately drawn based on the data presented.

Reviewer #1: Partly

Reviewer #2: Partly

**********

2. Has the statistical analysis been performed appropriately and rigorously?

Reviewer #1: Yes

Reviewer #2: No

**********

3. Have the authors made all data underlying the findings in their manuscript fully available (please refer to the Data Availability Statement at the start of the manuscript PDF file)?

The PLOS Data policy requires authors to make all data underlying the findings described in their manuscript fully available without restriction, with rare exception. The data should be provided as part of the manuscript or its supporting information, or deposited to a public repository. For example, in addition to summary statistics, the data points behind means, medians and variance measures should be available. If there are restrictions on publicly sharing data—e.g. participant privacy or use of data from a third party—those must be specified.

Reviewer #1: Yes

Reviewer #2: Yes

**********

4. Is the manuscript presented in an intelligible fashion and written in standard English?

PLOS Global Public Health does not copyedit accepted manuscripts, so the language in submitted articles must be clear, correct, and unambiguous. Any typographical or grammatical errors should be corrected at revision, so please note any specific errors here.

Reviewer #1: Yes

Reviewer #2: Yes

**********

5. Review Comments to the Author

Please use the space provided to explain your answers to the questions above. You may also include additional comments for the author, including concerns about dual publication, research ethics, or publication ethics. (Please upload your review as an attachment if it exceeds 20,000 characters)

Reviewer #1: This manuscript examines associations between ambient temperature and cardiovascular hospitalizations in Brazil (2010–2024) using multi-city time-series and a two-stage DLNM/meta-analysis framework. The authors report higher risk at colder temperatures (≤19 °C), neutral effects ~19–24 °C, and decreasing risk at higher temperatures. They also compare “tropical” vs “temperate” regions and find no significant differences. The topic is important and the two-stage DLNM approach is appropriate in principle. However, issues around outcome labeling (“incidence”), denominator/rate construction, city inclusion criteria, modeling choices (lag length; time-spline df), climate classification, and data availability must be addressed. I recommend major revision focused on (i) correcting the descriptive epidemiology and terminology, (ii) strengthening and documenting the modeling choices with sensitivity analyses, and (iii) clarifying data/selection procedures. Please see below my comments:

1) Throughout, the paper calls the outcome “incidence,” but the data are hospital admissions, not incident disease. Please replace “incidence” with “hospitalization rates/counts” everywhere, including title, abstract, figures, and discussion. (e.g., Abstract; Methods; mapping by state). Using “incidence per 100,000” with hospitalizations is potentially misleading to readers.

2) Excluding any city with fewer events than number of observation days (5,296) is unusual and may exclude many smaller cities/capitals, biasing toward large urban centers (Exclusion criteria). Consider alternative strategies (e.g., weekly aggregation, borrowing strength via hierarchical modeling, or keeping cities and letting the multivariate meta-analysis shrink unstable estimates). At minimum, justify this threshold, quantify how many cities were dropped by region, and assess sensitivity to this rule.

3) The spline for long-term/seasonal control is specified as ns(time, df = 70) “equivalent to 10 per year” (Appendix). Over 2010–2024 (~14 years), 10 df/year would be ~140 df, not 70. Please correct the description and justify the chosen df/year with references or AIC/QAIC diagnostics. Also report checks for residual autocorrelation/overdispersion and the overdispersion parameter.

4) Knot placement: exposure knots are “equally spaced over each city’s temperature range.” Consider standard practice (e.g., percentiles of the pooled temperature distribution) and report the centering temperature (e.g., MMT) used to compute RRs.

5) The model adjusts for DOW and a time spline, but no control for relative humidity, holidays, or air pollution—important confounders/effect modifiers in cardiovascular admissions studies. Even if not available nationwide, please conduct sensitivity analyses (where data exist) or justify omission. The Discussion acknowledges humidity/pollution but the model does not.

6) How were cities assigned to “tropical” vs “temperate” zones? Many states span multiple climate subtypes. Please specify classification source and rule, and show a table of city-level climate assignments. Also, the North region’s pooled analysis excludes Belém (Methods) without justification—please explain and provide sensitivity including Belém.

7) The primary pooled curve suggests decreasing RR at higher temperatures (Figure 3). Before interpreting as “protective,” please:

• show city-specific and region-specific exposure–response curves and heterogeneity (I², ²),

• explore different centering/MMT choices,

• extend lags, and

• assess potential exposure misclassification (station coverage/representativeness, urban heat island, station relocations).

These steps will help distinguish a truly protective effect from modeling or exposure biases.

8) The Data Availability statement says “Raw data are available upon reasonable request”, which is incompatible with PLOS policies that require open, unrestricted access to underlying data (with rare exceptions). Please deposit de-identified, aggregate daily counts and the analysis code (R scripts) in a public repository (e.g., Zenodo/OSF) and update the statement accordingly.

9) Describe how hourly station data became a city-level daily mean (station selection radius, multiple stations averaged, handling missing hours/days, QC flags).

10) Provide the exact crossbasis specification (knots/locations) and meta-analysis model formula, including moderators (climate type). Report heterogeneity statistics and (ideally) leave-one-out sensitivity.

11) Improve resolution; add axes labels/units (°C on x-axis, RR on y-axis), show tick marks, and consider 2-panel figures for pooled + regions with the same y-scale for comparability.

12) Add a table listing included 103 cities, population, period coverage, number of events, mean temperature, and assigned climate type.

13) Clarify selection bias from excluding most cities; discuss exposure misclassification (station siting/relocations; representativeness for large urban areas); acknowledge lack of adjustment for humidity/pollution/holidays and for age structure differences when presenting descriptive rates.

Reviewer #2: The manuscript requires substantial revision. The methodology section is insufficiently developed, as it omits critical components such as the study design, study setting, target population, sample size, sampling methods, and details of the statistical analysis. Furthermore, there is a clear discrepancy between the described methodology—particularly the statistical analysis—and the way the results are presented. In addition, the manuscript lacks a conclusion, which is essential for summarizing the findings and their implications.

See More detailed comments in the attached reviewed document.

**********

6. PLOS authors have the option to publish the peer review history of their article (what does this mean?). If published, this will include your full peer review and any attached files.

**Do you want your identity to be public for this peer review?**  If you choose “no”, your identity will remain anonymous but your review may still be made public.

For information about this choice, including consent withdrawal, please see our Privacy Policy.

Reviewer #1: No

Reviewer #2: **Yes:**  Anna Alfeus

**********

[NOTE: If reviewer comments were submitted as an attachment file, they will be attached to this email and accessible via the submission site. Please log into your account, locate the manuscript record, and check for the action link "View Attachments". If this link does not appear, there are no attachment files.]

-->Figure Resubmissions:

While revising your submission, we strongly recommend that you use PLOS’s NAAS tool (https://ngplosjournals.pagemajik.ai/artanalysis) to test your figure files. NAAS can convert your figure files to the TIFF file type and meet basic requirements (such as print size, resolution), or provide you with a report on issues that do not meet our requirements and that NAAS cannot fix.

After uploading your figures to PLOS’s NAAS tool - https://ngplosjournals.pagemajik.ai/artanalysis, NAAS will process the files provided and display the results in the "Uploaded Files" section of the page as the processing is complete. If the uploaded figures meet our requirements (or NAAS is able to fix the files to meet our requirements), the figure will be marked as "fixed" above. If NAAS is unable to fix the files, a red "failed" label will appear above. When NAAS has confirmed that the figure files meet our requirements, please download the file via the download option, and include these NAAS processed figure files when submitting your revised manuscript.

Attachment Submitted filename: PGPH-D-25-02810.pdf

# Author comment: Author response to Decision Letter 1

**DOI:** 10.1371/journal.pgph.0005294.r002

24 Mar 2026

Attachment Submitted filename: Response to Reviewers.docx

# Editor report: Decision Letter 1

**DOI:** 10.1371/journal.pgph.0005294.r003

**Authors:** Collins Asweto

26 Mar 2026

PGPH-D-25-02810R1

ASSOCIATION BETWEEN CLIMATIC VARIABLES AND CARDIOVASCULAR HOSPITALIZATIONS IN BRAZIL: AN ECOLOGICAL STUDY

PLOS Global Public Health

Dear Bruna,

Thank you for submitting your manuscript to PLOS Global Public Health. After careful consideration, we feel that it has merit but does not fully meet PLOS Global Public Health’s publication criteria as it currently stands. Therefore, we invite you to submit a revised version of the manuscript that addresses the points raised during the review process.

Please submit your revised manuscript by 26th May, 2026. If you will need more time than this to complete your revisions, please reply to this message or contact the journal office at globalpubhealth@plos.org. When you're ready to submit your revision, log on to https://www.editorialmanager.com/pgph/ and select the 'Submissions Needing Revision' folder to locate your manuscript file.

Please include the following items when submitting your revised manuscript:

- A letter that responds to each point raised by the editor and reviewer(s). You should upload this letter as a separate file labeled 'Response to Reviewers'.
- A marked-up copy of your manuscript that highlights changes made to the original version. You should upload this as a separate file labeled 'Revised Manuscript with Track Changes'.
- An unmarked version of your revised paper without tracked changes. You should upload this as a separate file labeled 'Manuscript'.

Guidelines for resubmitting your figure files are available below the reviewer comments at the end of this letter.

As the corresponding author, your ORCID iD is verified in the submission system and will appear in the published article. PLOS supports the use of ORCID, and we encourage all coauthors to register for an ORCID iD and use it as well. Please encourage your coauthors to verify their ORCID iD within the submission system before final acceptance, as unverified ORCID iDs will not appear in the published article. *Only*  the individual author can complete the verification step; PLOS staff *cannot*  verify ORCID iDs on behalf of authors.

We look forward to receiving your revised manuscript.

Kind regards,

Collins Otieno Asweto, PhD

Academic Editor

PLOS Global Public Health

Journal Requirements:

1. If the reviewer comments include a recommendation to cite specific previously published works, please review and evaluate these publications to determine whether they are relevant and should be cited. There is no requirement to cite these works unless the editor has indicated otherwise.

Reviewers' comments:

Figure Resubmissions:

-->While revising your submission, we strongly recommend that you use PLOS’s NAAS tool (https://ngplosjournals.pagemajik.ai/artanalysis) to test your figure files. NAAS can convert your figure files to the TIFF file type and meet basic requirements (such as print size, resolution), or provide you with a report on issues that do not meet our requirements and that NAAS cannot fix.

After uploading your figures to PLOS’s NAAS tool - https://ngplosjournals.pagemajik.ai/artanalysis, NAAS will process the files provided and display the results in the "Uploaded Files" section of the page as the processing is complete. If the uploaded figures meet our requirements (or NAAS is able to fix the files to meet our requirements), the figure will be marked as "fixed" above. If NAAS is unable to fix the files, a red "failed" label will appear above. When NAAS has confirmed that the figure files meet our requirements, please download the file via the download option, and include these NAAS processed figure files when submitting your revised manuscript.

# Author comment: Author response to Decision Letter 2

**DOI:** 10.1371/journal.pgph.0005294.r004

6 Apr 2026

Attachment Submitted filename: Response_to_Reviewers_auresp_2.docx

# Peer review: Decision Letter 2

**DOI:** 10.1371/journal.pgph.0005294.r005

**Authors:** Julia Robinson

8 Jul 2026

ASSOCIATION BETWEEN CLIMATIC VARIABLES AND CARDIOVASCULAR HOSPITALIZATIONS IN BRAZIL: AN ECOLOGICAL STUDY

PGPH-D-25-02810R2

Dear Dr. Dóris,

We are pleased to inform you that your manuscript 'ASSOCIATION BETWEEN CLIMATIC VARIABLES AND CARDIOVASCULAR HOSPITALIZATIONS IN BRAZIL: AN ECOLOGICAL STUDY' has been provisionally accepted for publication in PLOS Global Public Health.

Before your manuscript can be formally accepted you will need to complete some formatting changes, which you will receive in a follow up email. A member of our team will be in touch with a set of requests.

Please note that your manuscript will not be scheduled for publication until you have made the required changes, so a swift response is appreciated.

IMPORTANT: The editorial review process is now complete. PLOS will only permit corrections to spelling, formatting or significant scientific errors from this point onwards. Requests for major changes, or any which affect the scientific understanding of your work, will cause delays to the publication date of your manuscript.

If your institution or institutions have a press office, please notify them about your upcoming paper to help maximize its impact. If they'll be preparing press materials, please inform our press team as soon as possible -- no later than 48 hours after receiving the formal acceptance. Your manuscript will remain under strict press embargo until 2 pm Eastern Time on the date of publication. For more information, please contact globalpubhealth@plos.org.

Thank you again for supporting Open Access publishing; we are looking forward to publishing your work in PLOS Global Public Health.

Best regards,

Julia Robinson

Executive Editor

PLOS Global Public Health

***********************************************************

Reviewer Comments (if any, and for reference):

Reviewer's Responses to Questions

**Comments to the Author**

1. If the authors have adequately addressed your comments raised in a previous round of review and you feel that this manuscript is now acceptable for publication, you may indicate that here to bypass the “Comments to the Author” section, enter your conflict of interest statement in the “Confidential to Editor” section, and submit your "Accept" recommendation.

Reviewer #3: All comments have been addressed

**********

2. Does this manuscript meet PLOS Global Public Health’s publication criteria? Is the manuscript technically sound, and do the data support the conclusions? The manuscript must describe methodologically and ethically rigorous research with conclusions that are appropriately drawn based on the data presented.

Reviewer #3: (No Response)

**********

3. Has the statistical analysis been performed appropriately and rigorously?

Reviewer #3: (No Response)

**********

4. Have the authors made all data underlying the findings in their manuscript fully available (please refer to the Data Availability Statement at the start of the manuscript PDF file)?

The PLOS Data policy requires authors to make all data underlying the findings described in their manuscript fully available without restriction, with rare exception. The data should be provided as part of the manuscript or its supporting information, or deposited to a public repository. For example, in addition to summary statistics, the data points behind means, medians and variance measures should be available. If there are restrictions on publicly sharing data—e.g. participant privacy or use of data from a third party—those must be specified.

Reviewer #3: (No Response)

**********

5. Is the manuscript presented in an intelligible fashion and written in standard English?

PLOS Global Public Health does not copyedit accepted manuscripts, so the language in submitted articles must be clear, correct, and unambiguous. Any typographical or grammatical errors should be corrected at revision, so please note any specific errors here.

Reviewer #3: (No Response)

**********

6. Review Comments to the Author

Please use the space provided to explain your answers to the questions above. You may also include additional comments for the author, including concerns about dual publication, research ethics, or publication ethics. (Please upload your review as an attachment if it exceeds 20,000 characters)

Reviewer #3: A previous reviewer has already noted quite a few quantitative shortcomings such as the descriptive epidemiology and terminology, strengthening and documenting the modeling choices with sensitivity analyses, and clarifying data/selection procedures.

The threshold exclusions were a concern as well as diagnostic checks for df/yr and checks for over dispersion, and various modeling clarifications needed. In addition, standard two-stage analytical approach required clarification which the investigators also addressed. Text additions as well as tables sufficed to meet a number, if not most, of these concerns. Other review concerns dealt less stringently with the modeling corrections and presentation than with more general concerns.

Having reviewed the latest version it appears that the latest effort has cleaned up the statistical objections noted. The paper reads much better and the tables and figures are well formatted and interpretable. The conclusion follows from the revised document in that the investigators have had success in associating ambient temperature with hospitalization for cardiovascular diseases across more than 100 cities throughout the national territory.

**********

7. PLOS authors have the option to publish the peer review history of their article (what does this mean?). If published, this will include your full peer review and any attached files.

**Do you want your identity to be public for this peer review?**  If you choose “no”, your identity will remain anonymous but your review may still be made public.

For information about this choice, including consent withdrawal, please see our Privacy Policy.

Reviewer #3: No

**********
